# Lipopolysaccharide Attenuates Induction of Proallergic Cytokines, Thymic Stromal Lymphopoietin, and Interleukin 33 in Respiratory Epithelial Cells Stimulated with PolyI:C and Human Parechovirus

**DOI:** 10.3389/fimmu.2016.00440

**Published:** 2016-10-25

**Authors:** Tsang-Hsiung Lin, Chih-Chi Cheng, Hsing-Hao Su, Nan-Chieh Huang, Jih-Jung Chen, Hong-Yo Kang, Tsung-Hsien Chang

**Affiliations:** ^1^Graduate Institute of Clinical Medical Sciences, College of Medicine, Chang Gung University, Kaohsiung, Taiwan; ^2^Department of Medical Education and Research, Kaohsiung Veterans General Hospital, Kaohsiung, Taiwan; ^3^Department of Otorhinolaryngology – Head and Neck Surgery, Kaohsiung Veterans General Hospital, Kaohsiung, Taiwan; ^4^Department of Family Medicine, Zuoying Branch of Kaohsiung Armed Forces General Hospital, Kaohsiung, Taiwan; ^5^Department of Pharmacy, Tajen University, Pingtung, Taiwan; ^6^Department of Obstetrics and Gynecology, Center for Menopause and Reproductive Medicine Research, Kaohsiung Chang Gung Memorial Hospital, Kaohsiung, Taiwan; ^7^Department of Medical Laboratory Science and Biotechnology, Chung Hwa University of Medical Technology, Tainan, Taiwan

**Keywords:** TSLP, IL33, HMGB1, hygiene hypothesis, innate immunity

## Abstract

Epidemiological studies based on the “hygiene hypothesis” declare that the level of childhood exposure to environmental microbial products is inversely related to the incidence of allergic diseases in later life. Multiple types of immune cell-mediated immune regulation networks support the hygiene hypothesis. Epithelial cells are the first line of response to microbial products in the environment and bridge the innate and adaptive immune systems; however, their role in the hygiene hypothesis is unknown. To demonstrate the hygiene hypothesis in airway epithelial cells, we examined the effect of lipopolysaccharide (LPS; toll-like receptor 4 ligand) on the expression of the proallergic cytokines thymic stromal lymphopoietin (TSLP) and interleukin 33 (IL33) in H292 cells (pulmonary mucoepidermoid carcinoma cells). Stimulation with the TLR ligand polyI:C and human parechovirus type 1 (HPeV1) but not LPS-induced TSLP and IL33 through interferon regulatory factor 3 (IRF3) and NF-κB activity, which was further validated by using inhibitors (dexamethasone and Bay 11-7082) and short hairpin RNA-mediated gene knockdown. Importantly, polyI:C and HPeV1-stimulated TSLP and IL33 induction was reduced by LPS treatment by attenuating TANK-binding kinase 1, IRF3, and NF-κB activation. Interestingly, the basal mRNA levels of TLR signaling proteins were downregulated with long-term LPS treatment of H292 cells, which suggests that such long-term exposure modulates the expression of innate immunity signaling molecules in airway epithelial cells to mitigate the allergic response. In contrast to the effects of LPS treatment, the alarmin high-mobility group protein B1 acts in synergy with polyI:C to promote TSLP and IL33 expression. Our data support part of the hygiene hypothesis in airway epithelia cells *in vitro*. In addition to therapeutic targeting of TSLP and IL33, local application of non-pathogenic LPS may be a rational strategy to prevent allergies.

## Introduction

The hygiene hypothesis declares that a lack of early childhood exposure to environmental microorganisms and pathogens increases susceptibility to allergic diseases by suppressing the establishment of immune tolerance (1). Epidemiological data and experimental evidence showed that exposure to environmental pathogen-associated molecular patterns (PAMPs), such as lipopolysaccharide (LPS), are associated with decreasing the incidence of allergic diseases in later life (2–4). Similarly, muramic acid, a constituent of peptidoglycan of bacteria in the environment was found inversely associated with respiratory wheezing in rural school children (5). Those reports suggest that environmental microbes modulate allergic response.

Innate immunity is a rapid host defense response against invading pathogens, this response is essential to establish antigen-specific adaptive immunity to further eradicate pathogens and instruct the immune memory (6). Human epithelial cells form the largest primary physical barrier against environmental microbes and provide protection to the host via TLR-mediated responses of innate immunity (7–9). TLR signaling in skin and airway epithelial cells promotes the expression of proallergic cytokines, such as thymic stromal lymphopoietin (TSLP), granulocyte-macrophage colony-stimulating factor, interleukin-25 (IL25), and IL33, which are crucial for the initiation of the Th2 allergic immune cascade (7, 10–14). Epithelial cells command the innate and adaptive immune responses in atopic diseases (15). The implication of epithelial cells expressing the proallergic cytokines TSLP and IL33 in hygiene hypothesis is unknown.

Childhood exposure to environmental microorganism, such as viral infection can exacerbate asthma severity. Viral infection with rhinovirus, human metapneumovirus and respiratory syncytial virus can induce TSLP expression in airway epithelial cells (9, 16, 17). In particular, rhinovirus infection has been found associated with TSLP levels in the airways of young children (18). Rhinovirus infection can induce IL33 secretion in human bronchial epithelial cells to promote Th2 inflammation and exacerbate asthma severity in patients (19). This finding agreed with the observation of higher IL33 levels in the sera of patients with allergic rhinitis than normal controls (20). In addition, human parechovirus (HPeV), a small, round-structured, non-enveloped virus with a single-stranded and positive-sense RNA genome, belongs to the *Picornaviridae* (21). Nosocomial infection or outbreaks in neonate hospital departments seem to play a large role in HPeV infection (22, 23). Similar to rhinovirus, HPeV also causes respiratory disease in children, with high prevalence (24). It would be interested to understand whether HPeV1 acts like rhinovirus on prompting allergy.

Virus infection also can increase and activate TLR3 signal pathway (25). Among the various TLR ligands, only polyI:C (double-stranded RNA, TLR3 ligand) can stimulate high levels of TSLP expression, which is enhanced by the addition of IL4, IL13, or tumor necrosis factor α (26). Other TLR ligands, such as LPS (TLR4 ligand), CpG (TLR9 ligand), Pam_3_CSK_4_ (TLR2 ligand), and flagellin (TLR5 ligand), failed to induce TSLP expression in epithelial cells (16, 26). Similarly, IL33 mRNA expression could be induced by IFN-γ, the TLR9 ligand ODN2006, or polyI:C but not LPS in human nasal epithelial cells with allergic rhinitis (20, 27).

The immunoregulatory effect of the LPS/TLR4 axis in immune cells, such as dendritic cells and myeloid-derived suppressor cells was revealed in an animal model of asthma, which suggested that the dose of LPS is critical for the T helper 1 (Th1)/Th2 cell balance. Increased doses of LPS and antigens induce Th1 responses and inhibit allergic inflammation; however, reduced doses of LPS induce Th2 responses and promote airway inflammation (28–31). In addition to LPS, the TLR2 ligand Pam_3_CSK_4_ blocks the development of asthma (32). Therefore, TLRs in immune cells play roles during allergic airway responses.

The LPS failure to induce expression of TSLP and IL-33 prompted us to explore the mechanism by which LPS downregulates allergic cytokine production in response to polyI:C stimulation in airway epithelial cells. We established an *in vitro* model of the hygiene hypothesis in human airway epithelial mucoepidermoid pulmonary carcinoma cells (H292 cells) and used polyI:C treatment to mimic double-stranded viral RNA during replication to trigger inflammation (15, 26). We used our previously isolated and characterized clinical virus isolate, HPeV1 (33), to address whether LPS regulates virus-mediated allergic inflammation. The effects of LPS on polyI:C- and HPeV1-stimulated TSLP and IL33 mRNA expression were measured. Mechanistically, we also examined how LPS signaling subverts the polyI:C and HPeV1 signal axis in airway epithelial cells.

The non-histone nuclear protein high-mobility group protein B1 (HMGB1) is a damage-associated molecular pattern (DAMP) or called alarmin, which is released outside of the cells while cell activation, injury, or death (34). The HMGB1-mediated airway inflammation disease was characterized in the clinical and experimental asthma (35). In addition, HMGB1 from airway epithelial cells with respiratory syncytial virus infection primes epithelial cells and monocytes to inflammation stimuli in the airway (36). Multiple receptors were identified to be interacted with HMGB1, such as the receptor of advanced glycation end products (RAGE) or integrins, etc. (34). In addition, HMGB1 may act as an endogenous TLR2/4 ligand to trigger inflammatory responses (34, 37, 38). Thus, in this study, we also investigated whether HMGB1 regulates the TSLP and IL33 expression in polyI:C-stimulated airway epithelial cells.

## Materials and Methods

### Cells

The human mucoepidermoid pulmonary carcinoma cell line NCI-H292 (BCRC, 60732) was cultured in RPMI1640 medium (Invitrogen, Carlsbad, CA, USA) supplemented with 10% fetal bovine serum (FBS; Invitrogen) and 1% penicillin/streptomycin (Invitrogen) at 37°C in a 5% CO_2_ atmosphere. The human bronchial epithelial cell line NL-20 (ATCC-CRL-2503) was cultured in Ham’s F12 medium (Invitrogen, Carlsbad, CA, USA) with 10 ng/ml epidermal growth factor, 0.001 mg/ml transferrin, 500 ng/ml hydrocortisone, and 4% FBS and 1% penicillin/streptomycin (Invitrogen) at 37°C in a 5% CO_2_ atmosphere. A549 human lung epithelial carcinoma cells (ATCC: CCL-185), WS1 human fetal skin normal fibroblasts (BCRC: 60300), and HEK-293T cells (ATCC: CRL-3216) were cultured in DMEM supplemented with 10% fetal bovine serum and 1% penicillin/streptomycin at 37°C in a 5% CO_2_ atmosphere. The human primary nasal epithelium from three donors was isolated and cultured according to the previous report (39). The study was approved by the Institutional Review Board of Kaohsiung Veterans General Hospital (Protocol number: VGHKS98-VT8-06) and conformed to the current ethical principles of the Declaration of Helsinki. Written informed consents were obtained from all donors.

### Reagents

The double-stranded RNA, polyI:C, from Sigma-Aldrich (#P1530) and InvivoGen (#tlrl-picw) were used. Our testing results showed that the two polyI:C products of polyI:C have the similar activity of TSLP induction in H292 cells (Figure S1A in Supplementary Material). So, in this study, the polyI:C from Sigma-Aldrich was used.

Lipopolysaccharide from *Escherichia coli* 0111:B4 (#L2630), *Escherichia coli* 055:B5 (#L2880), *Escherichia coli* 026:B6 (#L-8274), *Escherichia coli* 0127:B8 (#L4516), *Klebsiella pneumoniae* (L4268), *Salmonella enterica serotype enteritidis* (#L7770), *Salmonella enterica serotype minnesota* (#L6261), *Pseudomonas aeruginosa 10* (#L9143), dexamethasone (#D4902), and Bay 11-7082 (#B5556) were all from Sigma-Aldrich (St. Louis, MO, USA) (40). We tested these eight different LPS in the TSLP and IL33 induction. Except the LPS from *Escherichia coli* 0127:B8, other seven types of LPS were not able to promote TSLP and IL33 expression; moreover, the polyI:C-induced TSLP and IL33 expression were attenuated by all of the tested LPS (Figures S1B,C in Supplementary Material). Based on the results of statistical analysis, the LPS from *Escherichia coli 0111:B4* was chosen in this study.

Recombinant human IL4 and HMGB1 were from Peprotech and R&D system, respectively (Rocky Hill, NJ, USA and Minneapolis, MN, USA). The expression vectors of IRF3, IRFs 5D, and IRF3 5A were described in our previous report (41). TurboFect transfection reagent (Thermo Scientific) was used for transient transfection following the manufacturer’s protocol.

### HPeV1 and Virus Titration

The strains of HPeV1 KVP6 (accession no. KC769584) were isolated by the Virology Group, Department of Microbiology, Kaohsiung Veterans General Hospital and propagated in Vero cells (ATCC: CCL-81) (33). To determine the virus titers, culture medium from HPeV1-infected cells were harvested for plaque-forming assays. Various virus dilutions were added to 6-well plates with 80% confluent Vero cells and incubated at 37°C for 2 h. After adsorption, cells were gently washed and overlaid with 1% agarose containing MEM supplemented with FBS. After 7 days’ incubation at 37°C, cells were fixed with 10% formaldehyde, then stained with 1% crystal violet for further plaque counting.

### Treatment

The treatment condition of polyI:C and IL4 or HPeV1 infection was evaluated with the TSLP induction in various cell types. H292 cells (1 × 10^6^) in 6-well plates were either transfected by 2.5 μg polyI:C with TurboFect or directly incubated with 30 μg/ml polyI:C-contained medium. The TSLP mRNA level was highly induced at 3 h post-stimulation and then declined at 6 h in both treatments; particularly, polyI:C transfection showed greater induction level of TSLP than just adding polyI:C (Figure S2A in Supplementary Material). Therefore, the method of polyI:C (2.5 μg) transfection was set for the allergic cytokine induction in this study. This treatment condition was confirmed in another cell types in A549 cells, which TSLP mRNA was promoted with polyI:C stimulation at 3 h, 6 h, and 12 h, and then declined at 24 h (Figure S2B in Supplementary Material). Similar results were observed in the NL20 cells (Figure S3A in Supplementary Material). The TSLP protein level was detected by immunoblotting, which showed the increased protein level of TSLP in H292 cells at 3–6 h and in A549 cells at 2–3 h after polyI:C transfection; then, the TSLP protein level was decreased in later time points (Figure S2C in Supplementary Material).

The TSLP induction by different concentration of recombinant IL4 was measured in H292 cells. Only the concentration of 20 ng/ml, but not 1 and 10 ng/ml, of IL4 was able to induce TSLP, the level was peaked at 3 h post-stimulation (Figure S2D in Supplementary Material). The TSLP induction by 20 ng/ml of IL4 had also detected in WS1 human fetal skin normal fibroblasts with a time course-dependent manner (Figure S2E in Supplementary Material).

HPeV1 infection-mediated innate immune activation in A549 cells was revealed in our previous report (33). Here, we also found TSLP induction in A549 cells infected with HPeV1 at multiplicity of infection (MOI) = 5. Two induction peaks were observed at 2 and 36 h post infection (hpi) (Figure S2F in Supplementary Material).

Before LPS treatment, the cell culture medium was replaced with serum-free RPMI medium for 1 h, and then various concentrations of LPS were added to the cells and incubated for 2 h for short-term LPS treatment. LPS-treated cells were stimulated with polyI:C or infected with HPeV1 for the indicated times. In certain case, the cells were stimulated by recombinant IL4 (20 ng/ml) or HMGB1 (1 μg/ml). For long-term LPS treatment, H292 cells were incubated with LPS (30 μg/ml) for 8 or 16 days, or LPS (0.3 μg/ml) for 60 days, and the LPS-containing growth medium was refreshed every 2 days. Before polyI:C stimulation, long-term LPS-treated H292 cells were incubated with serum-free medium for 1 h followed by LPS treatment for 2 h, then stimulated with polyI:C for 3 h. In the inhibitor treatment group, dexamethasone or Bay 11-7082 was added to cells, and cells were incubated for 2 h before stimulation with polyI:C.

### Cell Proliferation Assay

WST-1 assay (Roche, Basel, Switzerland) was used to monitor cell proliferation (42, 43); H292 cells were trypsinized and resuspended in culture medium, then plated at 5 × 10^3^ cells per well in 96-well plates and incubated overnight. After LPS treatment followed by polyI:C transfection, cells were incubated with 10 μl WST-1 reagent for 2 h. The cell viability was quantified by multi-well spectrophotometry (Anthos, Biochrom, Cambridge, UK). The absorbance at 450 nm was monitored, and the reference wavelength was set at 620 nm.

### Quantitative Real-time PCR

Total RNA was extracted by using TRIzol reagent (Thermo Fisher Scientific) according to the manufacturer’s instructions. The cDNA was synthesized from 5 μg total RNA by using a Superscript III reverse transcriptase kit (Thermo Fisher Scientific) with oligo (dT) primers. Real-time PCR involved 10 ng total cDNA and SYBR green master mix (Applied Biosystems, Carlsbad, CA, USA) with the ABI StepOne Plus Real-Time PCR System (Applied Biosystems) (44). The primers for qPCR are in Table S1 in Supplementary Material. The relative mRNA level was normalized to that of glyceraldehyde-3-phosphate dehydrogenase (GADPH), as a loading control.

### Immunoblotting

Cells were lysed in 2% SDS buffer [2% SDS, 50 mM Tris–HCl (pH 7.5), 20 mM *N*-ethylmaleimide plus complete protease inhibitor cocktail and phosphatase inhibitor cocktail (Roche)]. Whole cell extracts (WCEs) were homogenized by sonication for 10 s with a sonicator (Soniprep 150, MSE, London, UK) (45). Protein concentrations were determined by *DC* Protein Assay (Bio-Rad). In total, 100 μg WCEs were separated by 10 or 12% SDS-PAGE, then transferred to polyvinylidene fluoride (PVDF) membranes (EMD Millipore, Billerica, MA, USA), incubated with primary antibody overnight at 4°C, then horseradish peroxidase-conjugated secondary antibody (Jackson ImmunoResearch Laboratory, West Grove, PA, USA) for 90 min and bands were detected by using the ECL reagent (Advasta, Menlo Park, CA, USA) with the BioSpectrum Image System (UVP, Upland, CA, USA). Protein or phosphor-protein levels were normalized to that of β-actin or corresponding total protein, respectively; and represented as fold changes compared with the control. The primary antibodies against interferon regulatory factor 3 (IRF3; #sc-9082), NF-κB p65 (#sc-372), and TSLP (#sc33791) were from Santa Cruz Biotechnology (Santa Cruz, CA, USA). Antibodies against RIG-I (#4520), IκBα (#4814), TBK-1 (#3504), phospho-TBK-1 (Ser172, #5483), and phospho-NF-κB p65 (Ser468, #3039 and Ser536, #3033) were from Cell Signaling (Danvers, MA, USA). Anti-phospho-IRF3 (#ab76493) and anti-β-actin (#MA5-15739) antibodies were from Abcam (Cambridge, UK) and Thermo Fisher Scientific (Waltham, MA, USA), respectively.

### NF-κB Luciferase Reporter Assay

Cells cultured in 12-well plates were transfected with NF-κB-Luc reporter plasmids (46) by TurboFect. pRL-TK (Promega), encoding *Renilla* luciferase under a herpes simplex virus thymidine kinase promoter, was an internal control. Twenty-four hours post-transfection, cells were stimulated with polyI:C. Cell lysates were collected for the dual-luciferase assay (Promega). Firefly luciferase activity was normalized relative to that of *Renilla* luciferase.

### Immunofluorescence Assay

H292 cells were fixed in 4% paraformadehyde for 20 min and permeabilized with 0.5% Triton X-100 for 15 min, washed three times with PBS, then incubated with 10% skim milk in PBS for 15 min to block non-specific antibody binding. To detect the cellular location of NF-κB p65 or IRF3, cells were incubated with antibodies against NF-κB p65 or IRF3 (1:500 in PBS) at 4°C overnight, then with the secondary antibody biotinylated goat anti-rabbit IgG (1:500 in PBS, Thermo Fisher Scientific) at room temperature for 90 min, then Alexa Fluor 568 streptavidin (1:500 in PBS, Thermo Fisher Scientific) for another 90 min at room temperature. Nuclear counterstaining involved staining with 4′,6-diami-dino-2-phenylindole (DAPI) for 10 min at room temperature. Fluorescence signals were observed by fluorescence microscopy (ZEISS, Observer A1, Oberkochen, Germany). Anti-HPeV VP0 antibody was used to detect HPeV1-infected H292 cells (33).

### NF-κB p65 and IRF3 Knockdown

Short hairpin RNA (shRNA) specific to NF-κB p65 and IRF3 was obtained from the National RNAi Core Facility, Institute of Molecular Biology/Genomic Research Center, Academia Sinica. HEK293T cells were transfected with shRNA lentivirus by using pMD.G, pCMV-ΔR8.91, PLKO.1 puro scramble control (shCtrl) or PLKO.1 puro shNF-κB p65 for 24 h, then culture medium was refreshed. Media containing lentivirus was harvested at 72 h post-transfection. H292 cells were infected with shNF-κB p65 or shCtrl lentivirus and infected cells were selected with puromycin (1 μg/ml) for 3 days.

### Statistical Analysis

Significant differences between groups were analyzed by two-tailed Student *t*-test with the software GraphPad Prism 5 (La Jolla, CA, USA). Data are presented as mean ± SD. *P* < 0.05 was considered statistically significant.

## Results

### LPS-Attenuated PolyI:C-Induced TSLP and IL-33 Expression and Cell Damage

**Figure 1:**
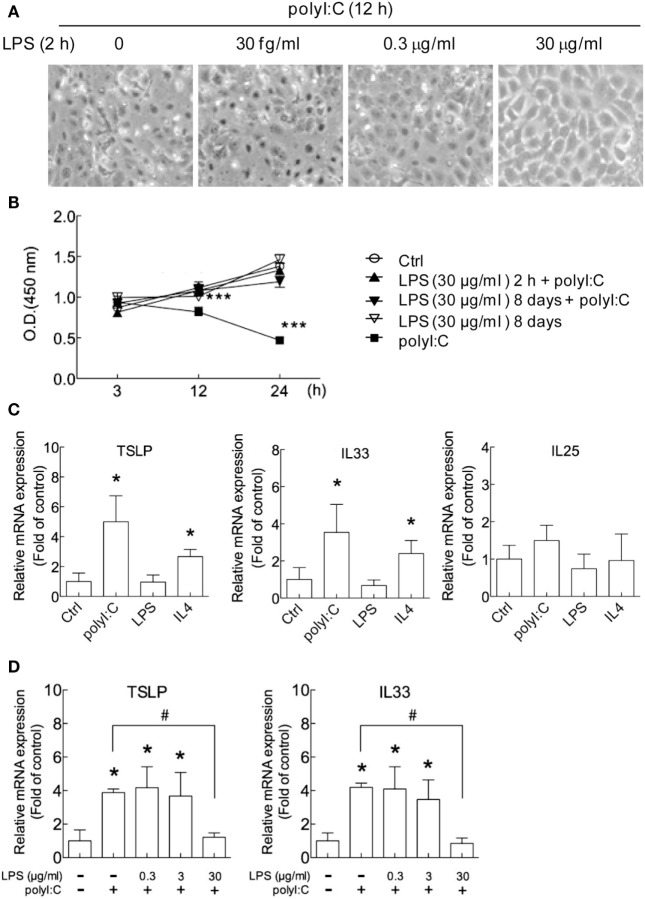
**PolyI:C-induced cell damage and allergic cytokine expression are attenuated by lipopolysaccharide (LPS) in airway epithelial cells**. **(A)** H292 cells were treated with LPS (3 × 10^−8^, 0.3 and 30 μg/ml) for 2 h followed by polyI:C (2.5 μg) transfection for 12 h. Representative images from three independent experiments show that cell morphology under an inverted microscope. **(B)** H292 cells were treated with control medium (Ctrl) or 30 μg/ml LPS for 2 h or 8 days then stimulated with polyI:C (100 ng) for 3, 12, or 24 h. Cell viability was analyzed by WST-1 assay. Results are representative of three independent experiments. **(C)** H292 cells were treated with polyI:C (2.5 μg), LPS (30 μg/ml), or IL4 (20 ng/ml) for 3 h, and thymic stromal lymphopoietin (TSLP), interleukin 33 (IL33) and IL25 mRNA expression was analyzed by RT-qPCR. **(D)** H292 cells were pretreated with LPS (0, 0.3, or 30 μg/ml) for 2 h, then stimulated with polyI:C for 3 h. The expression of TSLP and IL33 were monitored by RT-qPCR and normalized to the internal control GAPDH; fold induction over controls is presented. Data of RT-qPCR values are mean ± SD from three independent experiments. Two- tailed student *t*-test, *P < 0.05, ***P < 0.001 compared to controls; ^#^*P* < 0.05.

To investigate the hygiene hypothesis in airway epithelial cells *in vitro*, we used H292 cells because the gene expression profile of H292 airway epithelial cells is similar to that of primary nasal epithelial cells from healthy human controls on stimulation with house dust mite extracts (47). In studies of cell morphology, polyI:C stimulation induced damage in H292 cells at 12 h post-transfection (Figure 1A), which is similar to previous findings (48). We noted that LPS treatment for 2 h dose-dependently protected cells against death induced by polyI:C, with high-dose (30 μg/ml) but not low-dose (3 × 10^−8^ μg/ml) LPS conferring normal cell morphology (Figure 1A). PolyI:C-induced H292 cell death was inhibited by LPS treatment for 2 h or 8 days (Figure 1B). The data suggest a cross-regulation between TLR4 and TLR3 signaling pathway.

Gene expression of the allergic inflammation cytokines TSLP, IL33, and IL25 was measured in H292 cells. The TSLP and IL33 expression were significantly increased with polyI:C and IL4 treatment, by approximately three- to fivefold and twofold, respectively (Figure 1C) but not LPS. PolyI:C modestly increased IL25 expression, and IL4 has no effect (Figure 1C). PolyI:C and IL4-induced TSLP expression was also determined in A549 and WS-1 cells, respectively (Figures S2A–E in Supplementary Material). The polyI:C and IL4-induced TSLP and IL33 expression were also shown in NL20 cells, an immortalized, non-tumorigenic human bronchial epithelial cell line (Figures S3A–D in Supplementary Material). Low-dose LPS (0.3 and 3 μg/ml) had no significant effect on the polyI:C-stimulated mRNA level of TSLP and IL33, but high-dose LPS (30 μg/ml) suppressed the increased TSLP and IL33 level (Figure 1D). The reduction of polyI:C-induced TSLP expression by LPS with a dose-dependent manner was also showed in the human primary nasal epithelial cells (Figures S4A,B in Supplementary Material). These data suggest that LPS concentration is critical for its inhibitory effect.

### LPS Treatment Inhibits PolyI:C-Triggered IRF3 Activation

**Figure 2:**
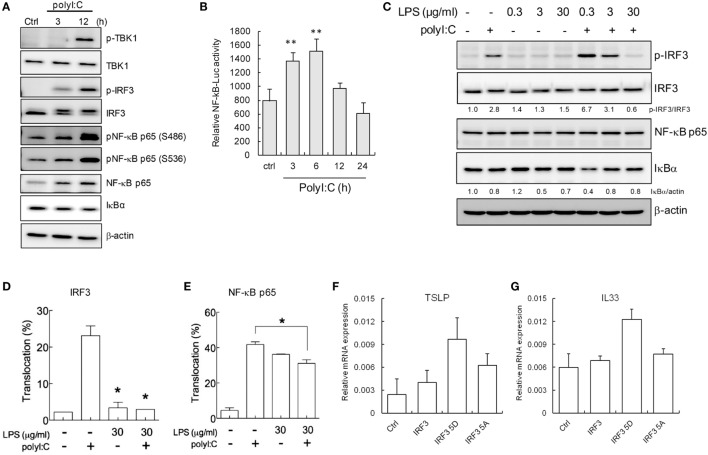
**LPS downregulates polyI:C-triggered interferon regulatory factor 3 (IRF3) and NF-κB activation**. **(A)** H292 cells were stimulated with polyI:C (2.5 μg) for 3 and 12 h. Total cell extracts underwent SDS-PAGE and immunoblotting with specific antibodies. Data shown are representative of three different experiments. **(B)** Dual-luciferase assay of NF-κB luciferase reporters. H292 cells transfected with Luc reporter (0.6 μg) and pRL-TK (0.06 μg) were treated with polyI:C (2.5 μg) for 3–24 h. Data are mean ± SD from three independent tests. **P ≤ 0.01 vs. untreated group. **(C)** H292 cells were pretreated with doses of LPS as indicated for 2 h and then stimulated with polyI:C (2.5 μg) for 3 h. Cell extracts underwent SDS-PAGE and immunoblotting with specific antibodies. The signaling proteins expression level was analyzed by density meter and normalized to untreated control. Data shown are representative of three different experiments. **(D,E)** IRF3 and NF-κB p65 nuclear translocation was measured by immunofluorescence assay in H292 cells treated with LPS (30 μg/ml) for 2 h, then polyI:C stimulation for 3 h. Nuclear translocation rate is shown as mean ± SD from three observed fields. *P < 0.05 compared to controls. **(F,G)** H292 cells were transfected with control vector, wild-type IRF3, IRF3 5D (constitutive active IRF3), and IRF3 5A (dominate negative IRF3) for 48 h. The expression of TSLP and IL33 mRNA were measured by RT-qPCR and normalized to the internal control GAPDH. Values represent the average of three independent experiments ± SD.

TLR-mediated innate immune responses are elegantly regulated by several adaptive signaling proteins and transcription factors. In the TLR3 signaling axis, activation of the transcription factors IRF3 and NF-κB p65/50 by I kappa B kinase (IKK) and IKK-related kinases, such as TBK-1, IKKϵ, IKKα, and IKKβ, is required for transcription of downstream cytokines (6, 49). In H292 cells, this pathway could be activated by polyI:C, the IRF3 phosphorylation was detected at 3 h after stimulation (Figure 2A). And the conspicuous increases of phospho-TBK1, -IRF3, and -NF-κB p65 (Ser456 and Ser536) were shown at 12 h (Figure 2A). The protein level of NF-κB p65 increasing and IκBα degradation was also noted (Figure 2A). The NF-κB activation by polyI:C was further validated by luciferase reporter assay (Figure 2B). Those data indicate the competent innate immunity in H292 cells.

To determine the mechanism why LPS-attenuated polyI:C-induced TSLP and IL-33 expression, IRF3, and NF-κB were investigated, PolyI:C-induced IRF3 phosphorylation was inhibited by high-dose (30 μg/ml) but not low-dose LPS treatment in H292 cells (Figure 2C). By contrast, the lowest dose of LPS (0.3 μg/ml) enhanced polyI:C-mediated IRF3 phosphorylation approximately 2.4-fold as compared with polyI:C stimulation alone. PolyI:C stimulation induced an 11-fold decrease in IRF3 phosphorylation with 30 μg/ml LPS treatment as compared with 0.3 μg/ml LPS treatment (Figure 2C). Although the total NF-κB p65 level was not significantly changed, as compared with that observed with 0.3 μg/ml LPS treatment, 3 and 30 μg/ml LPS treatment blocked the polyI:C-induced IκBα degradation by approximately twofold, which suggests that the polyI:C-mediated activation of the NF-κB pathway was downregulated with high-dose LPS (Figure 2C). The nuclear translocation of IRF3 and NF-κB confirmed that LPS significantly interfered with polyI:C-induced IRF3 and NF-κB activation (Figures 2D,E). The representative immunofluorescence images are shown in Figure S5 in Supplementary Material. IRF3 activity-associated TSLP and IL33 expression was further validated by ectopic expression of constitutive active IRF3 (IRF3 5D) but not dominant negative IRF3 mutant (IRF3 5A) (Figures 2F,G).

### Blocking IRF3 or NF-κB Activation Inhibits TSLP and IL-33 Induction

**Figure 3:**
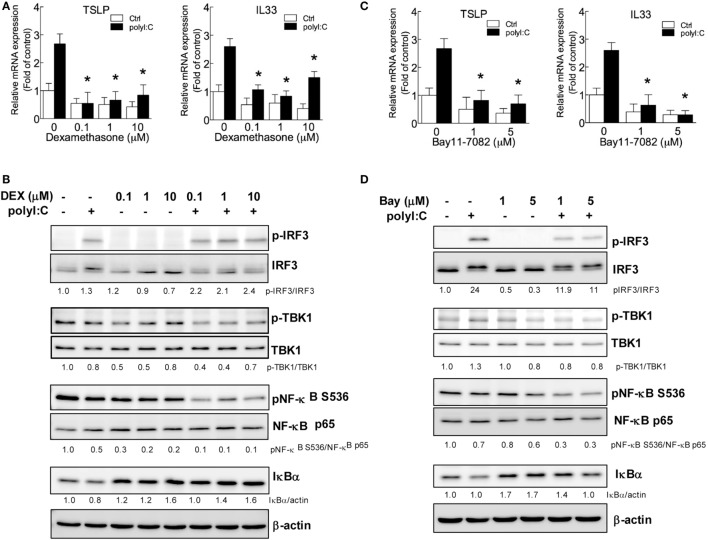
**IRF3 and NF-κB inhibitors suppress polyI:C-stimulated TSLP and IL33 expression**. H292 cells were treated with NF-κB or IRF3 inhibitors dexamethasone (0.1, 1, and 10 μM) or Bay 11-7082 (1 and 5 μM) for 2 h, then challenged with polyI:C (2.5 μg) for 3 h. **(A,C)**. The mRNA expression of TSLP and IL33 was measured by RT-qPCR. Data are mean ± SD from three independent experiments. *P < 0.05 compared to untreated controls with polyI:C stimulation. **(B,D)** Immunoblotting analysis with the indicated antibodies. Data shown are representative of three different experiments. The proteins expression level was measured by density meter; the value is normalized to untreated control.

Based on the findings in Figures 1 and 2, blocking IRF3 and NF-κB activity by LPS treatment might be the key mechanism underlying the hygiene hypothesis. We further validated the role of NF-κB and IRF3 in TSLP and IL33 induction by a pharmatheutical approach. The NF-κB inhibitor dexamethasone (50) significantly downregulated TSLP and IL33 mRNA expression in H292 cells stimulated with polyI:C (Figure 3A), and immunoblotting data confirmed the inhibitory effect of dexamethasone in interfering with NF-κB activity by inhibiting IκBα degradation (Figure 3B). Although phospho-IRF3 level was enhanced in polyI:C-stimulated cells with dexamethasone treatment (Figure 3B), this phenomenon did not alter the inhibitory activity of dexamethasone (Figure 3A). Bay 11-7082 is an IKK inhibitor that targets IKKα/β, TBK1, IRAK1/4, and TAK1 to modulate NF-κB and IRF3 activity (51). PolyI:C-stimulated TSLP and IL33 mRNA expression was inhibited in H292 cells treated with Bay 11-7082 (Figure 3C). Bay 11-7082 effectively inhibited polyI:C-induced IRF3 phosphorylation and IκBα degradation (Figure 3D).

**Figure 4:**
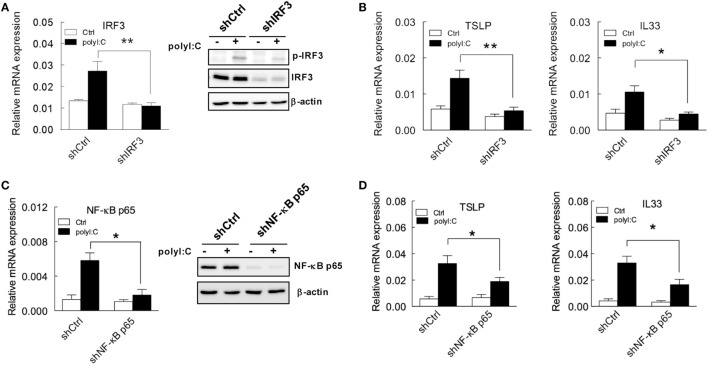
**TSLP and IL33 induction is inhibited in H292 cells with IRF3 and NF-κB p65 knockdown with polyI:C stimulation**. **(A,C)** H292 cells were infected with lentivirus carrying IRF3 and NFκB p65 shRNA. After puromycin selection, the mRNA and protein expression of IRF3 and NF-κB p65 was analyzed by RT-qPCR (left panel) and immunoblotting (right panel), respectively. Data shown are representative of three different experiments. **(B,D)** TSLP and IL33 mRNA expression was measured by RT-qPCR in H292 cells with IRF3 and NF-κB knockdown that were treated with polyI:C. Data are mean ± SD from three independent experiments. *P < 0.05, **P < 0.01.

The indispensable activity of IRF3 and NF-κB in TSLP and IL33 induction was further demonstrated by shRNA knockdown. IRF3 knockdown efficiency was confirmed by RT-qPCR, and immunoblotting showed that polyI:C failed to induce IRF3 protein expression and phosphorylation in H292 cells with IRF3 knockdown (Figure 4A). Importantly, polyI:C-induced TSLP and IL33 expression was inhibited with IRF3 knockdown (Figure 4B). The mRNA and protein levels of NF-κB p65 were not increased in knockdown cells with polyI:C stimulation (Figure 4C). In addition, polyI:C-induced TSLP and IL33 expression was inhibited with NF-κB p65 knockdown, by ~40 and 50%, respectively (Figure 4D). These data validated that the activity of IRF3 and NF-κB is associated with TSLP and IL33 induction by polyI:C.

### LPS-Attenuated HPeV1-Mediated TSLP and IL33 Expression and Signaling

**Figure 5:**
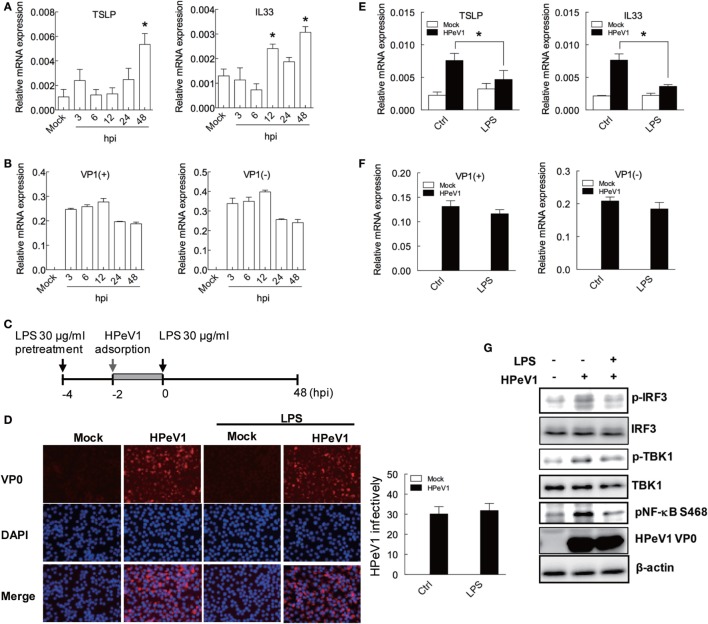
**LPS-attenuated HPeV1-mediated TSLP and IL33 expression and signaling**. **(A,B)** TSLP and IL33, HPeV1 VP1 (+), and HPeV1 VP1 (−) expression were measured in H292 cells with mock or HPeV1 infection at various times. Data are mean ± SD from three independent experiments. **(C)** The schematic shows LPS treatment and HPeV1 infection in H292 cells. **(D)** Left panel: H292 cells, pretreated or untreated with LPS, were subjected to HPeV1 infection at multiplicity of infection (MOI) = 2.5. HPeV1 infectivity was analyzed by immunofluorescence assay with anti-HPeV0 antibody. HPeV1-infected cells show red fluorescence, and DAPI staining (blue color) show cell nuclei. The merged images show overlapping anti-HPeV VP0 and DAPI staining. Results are representative of three independent experiments. Right panel: HPeV1 infectivity was calculated from three observation fields. **(E,G)** TSLP and IL33, HPeV1 VP1 (+) and HPeV1 VP1 (−) expression was measured in H292 cells with or without HPeV1 infection (Mock) and with (control medium) or without LPS treatment. **(F)** H292 cells were pretreated with or without LPS (30 μg/ml) then infected with or without HPeV1 (MOI = 2.5), then underwent immunoblotting with specific antibodies. Data are mean ±SD from three independent experiments. *P < 0.05 compared to controls.

To determine the TSLP and IL33 induction in H292 cells with viral infection, the cell was infected by HPeV1 for various times. The TSLP and IL33 levels were significantly induced at 48 hpi (Figure 5A). The expression of HPeV VP1 positive strain (+) and negative strain (−) genes showed HPeV1 replication in H292 cells (Figure 5B). HpeV1-induced TSLP expression was also shown in A549 cells (Figure S2F in Supplementary Material). To evaluate whether LPS regulates HPeV1 infection-mediated allergic inflammation, H292 cells were treated with LPS before HPeV1 infection (Figure 5C). Immunofluorescence assay showed no significant change of the HPeV1 infectivity in H292 cells with or without LPS treatment (Figure 5D); whereas, compare to untreated control cells, LPS significantly reduced TSLP and IL33 level in H292 cells infected with HPeV1 (Figure 5E). Again, the data of HPeV1 VP1 gene expression indicated no effect of LPS in HPeV1 replication (Figure 5F). Immunoblotting analysis showed that LPS downregulated the phosphorylation of IRF3, TBK1, and NF-κB in HPeV1-infected H292 cells (Figure 5G). Taken together, these data suggest that LPS modulates HPeV1 infection-triggered allergic cytokines expression.

### Long-term LPS Treatment Inhibits TSLP and IL33 Production Pathways

**Figure 6:**
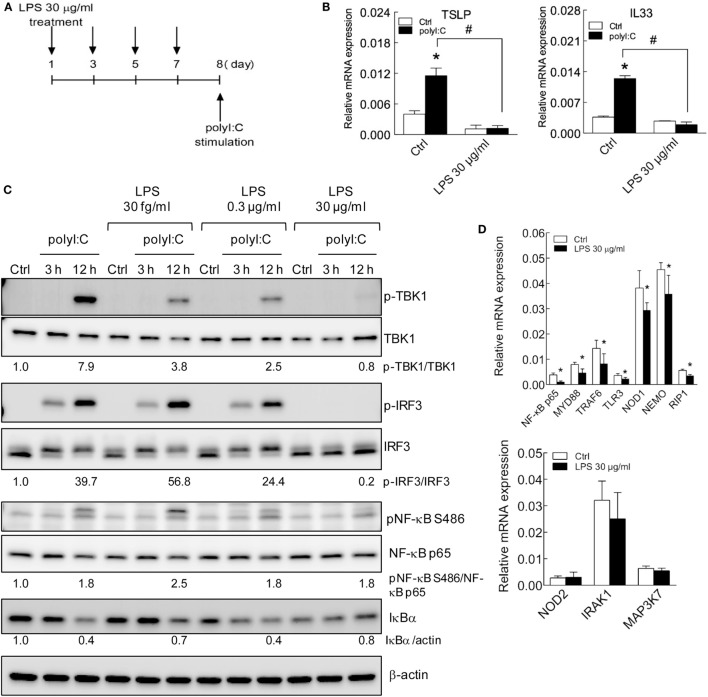
**TSLP and IL33 induction is impaired in H292 cells with long-term LPS exposure**. **(A)** The schematic shows long-term LPS treatment of H292 cells. H292 cells were stimulated with LPS 30 μg/ml for 8 days, then with polyI:C (2.5 μg) for 3 h. **(B)** RT-qPCR analysis of mRNA levels of polyI:C-induced TSLP and IL-33 in H292 cells with long-term LPS (30 μg/ml) treatment. **(C)** H292 cells were treated with various doses of LPS for 8 days, then stimulated with polyI:C (2.5 μg) for 3 h. Immunoblotting analysis with the indicated antibodies. The ratio of signaling protein expression was normalized to medium control. Results are representative of three independent experiments. **(D)** RT-qPCR analysis of TLR signaling gene expression in H292 cells with long-term LPS treatment. Data are mean ± SD from three independent experiments. *P < 0.05 compared to control; ^#^*P* < 0.05.

The short-term treatment with high level LPS showed inhibitory effect of allergic cytokines response in epithelial cells (Figures 1–5), whereas the effect of long-term treatment of LPS remained to be explored. Thus, to establish long-term LPS-treated H292 cells, cells were incubated with LPS (30 μg/ml) and subcultured every 2 days with LPS-containing medium. On day 8 of LPS treatment, cells were stimulated with polyI:C (Figure 6A). Similar to short-term LPS treatment, with long-term LPS treatment, with polyI:C, the increased mRNA expression of TSLP and IL33 was inhibited (Figure 6B). The polyI:C-induced TSLP protein level was inhibited by LPS treatment (Figure S6A in Supplementary Material). Furthermore, the protein levels of TLR3 downstream adaptor signaling proteins, phosphorylated TBK1, IRF3 were inhibited with high-dose (30 μg/ml), long-term LPS treatment and polyI:C stimulation for 3 and 12 h. The IκBα degradation was also downregulated by high-dose LPS treatment in H292 cells stimulated with polyI:C for 12 h (Figure 6C).

**Figure 7:**
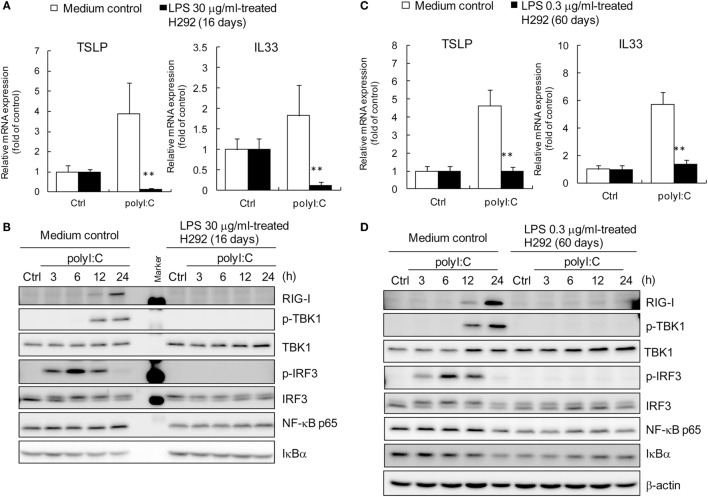
**Defective activation of RIG-I-IRF3 signaling axis in H292 cells with long-term LPS treatment**. LPS-trained H292 cells were established by long-term treatment of high level LPS (30 μg/ml) and low-level LPS (0.3 μg/ml) for 16 days **(A)** and 60 days **(C)**, respectively. RT-qPCR analysis of mRNA levels of polyI:C-induced TSLP and IL-33 are shown. The growth medium cultured cells were as the untreated control group. Data are mean ± SD from three independent experiments. **P < 0.01 compared to control. **(B,D)** The whole cell extracts from high level and low-level LPS-trained or untrained H292 cells were harvested at 3, 6, 12, and 24 h after polyI:C stimulation. Immunoblotting analysis was performed with the indicated antibodies. Results are representative of three independent experiments.

We also determined whether the basal mRNA level of signaling proteins was changed with long-term LPS stimulation. Compared with untreated control cells, cells with long-term LPS treatment showed significantly downregulated basal mRNA level of NF-κB p65, MyD88, TRAF6, TLR3, NOD1, NEMO, and RIP1 but not NOD2, IRAK1, or MAP3K7 (Figure 6D). Similar results were displayed in H292 cells with long-term LPS (30 μg/ml) treatment for 16 days that LPS decreased polyI:C-mediated TSLP and IL33 expression (Figure 7A). The polyI:C mediated-protein level of retinoic acid-inducible gene I (RIG-I, a dsRNA receptor), phosphorylated TBK1 and IRF3 were inhibited in H292 cells with LPS (30 μg/ml) treatment for 16 days (Figure 7B). Again, the basal mRNA level of singling protein genes were downregulated in those cells (Figure S6B in Supplementary Material). These data suggest a fundamental transcription modulation in LPS-treated cells. Consequently, in these cells, allergic cytokine expression was decreased after polyI:C stimulation.

Our data indicated H292 cells with high level (30 μg/ml) LPS treatment for short term (2 h) and long term (8 and 16 days) were able to modulate polyI:C-induced TSLP and IL33 expression; but low-level (0.3 μg/ml) LPS treatment for 2 h showed no effect against allergic inflammation.

To evaluate the effect of low-level LPS and long-term treated H292 cells on TSLP and IL33 expression, LPS-trained H292 cells were established via maintaining in low-level (0.3 μg/ml) LPS-containing growth medium for 60 days. The mRNA analysis showed that polyI:C failed to induced TSLP and IL33 in the LPS-trained H292 cells (Figure 7C). In addition, similar to the H292 cells with long-term high-level LPS treatment, the polyI:C-associated RIG-I, phosphorylated TBK1 and IRF3 expression was suppressed (Figure 7D).

### HMBG1 Acts in Synergy with PolyI:C to Induce TSLP and IL33 Expression

**Figure 8:**
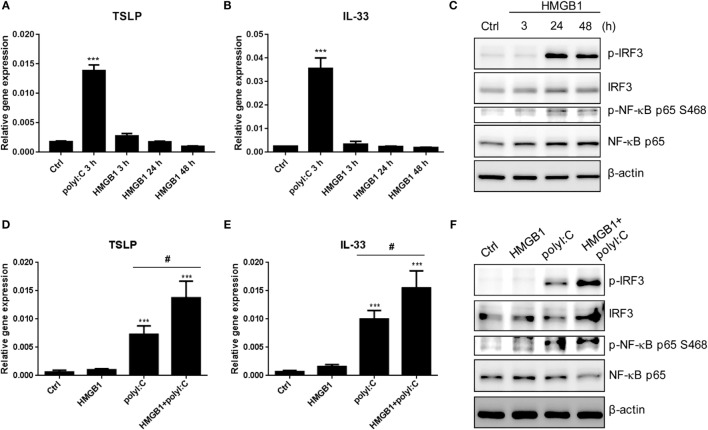
**HMGB1 enhances polyI:C-stimulated TSLP and IL33 expression**. **(A,B)** RT-qPCR analysis of TSLP and IL33 expression in H292 cells stimulated with polyI:C (2 μg) for 3 h or HMGB1 (1 μg/ml) for 3, 24, and 48 h. Data are mean ± SD from three independent experiments. ***P < 0.001 compared to control. **(C)** The whole cell extracts from H292 cells were harvested at 3, 24, and 48 h after HMGB1 (1 μg/ml) stimulation. Immunoblotting analysis was performed with the indicated antibodies. Results are representative of three independent experiments. **(D,E)** H292 cells were pretreated with HMGB1 (1 μg/ml) for 3 h, then stimulated with polyI:C for 3 h. The expression of TSLP and IL33 were monitored by RT-qPCR. Data are mean ± SD from three independent experiments. ***P < 0.001 compared to controls; ^#^*P* < 0.05. **(F)** H292 cells were pretreated with HMGB1 for 3 h and then stimulated with or without polyI:C (1 μg) for 3 h. Cell extracts underwent SDS-PAGE and immunoblotting with specific antibodies. Data shown are representative of three different experiments.

To evaluate the role of HMGB1 in proallergic cytokines expression, the TSLP and IL33 RNA were measured in the H292 cell with HMGB1 stimulation for 3, 24, and 48 h. The HMGB1 alone was not able to induced TSLP and IL33, while polyI:C showed positive induction (Figures 8A,B). Although the IRF3 and NF-κB were activated at 24 and 48 h after HMGB1 stimulation (Figure 8C); however, those signals might not be enough for downstream TSLP and IL33 expression. We further evaluated that whether HMGB1 plays a similar role to LPS to attenuate the activity of polyI:C-mediated proallergic cytokine expression. Surprisingly, in the polyI:C-stimulated cells, the RNA level of TSLP and IL-33 was increased by HMGB1 pretreatment for 3 h (Figures 8D,E). The immunoblotting also showed that HMGB1 enhanced the level of polyI:C-mediated IRF3 phosphorylation (Figure 8F). These results suggest that HMGB1 acts in synergy with polyI:C to promote TSLP and IL33 expression, which may be involved in allergic inflammation.

## Discussion

**Figure 9:**
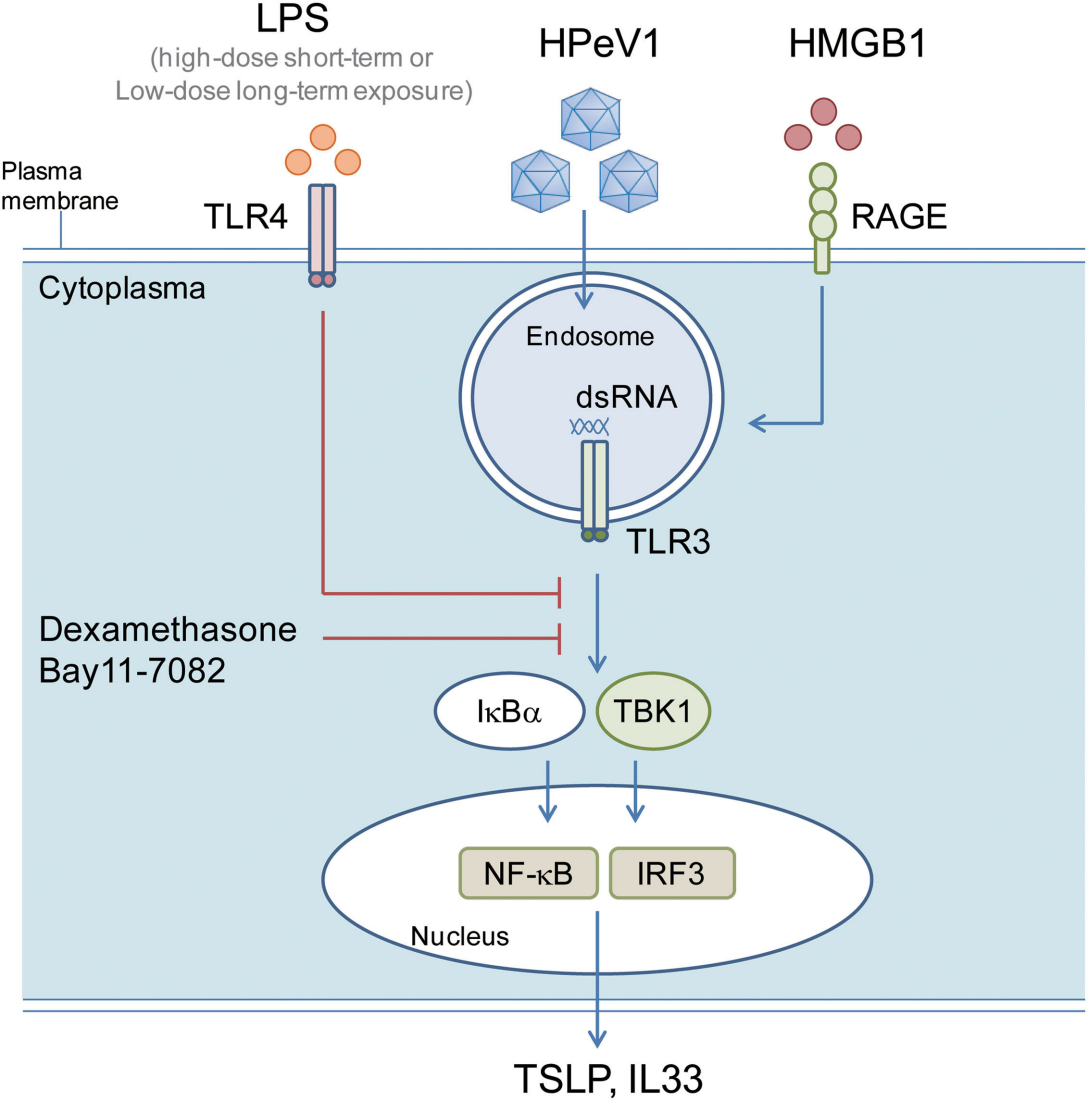
**LPS inhibits polyI:C- and virus-induced proallergic cytokines expression via targeting TBK1, IRF3, and NF-κB response**. A schematic model shows the possible mechanism of the LPS modulating TSLP and IL33 in airway epithelial cells. The polyI:C or HPeV1 activated TLR signaling is inhibited in the cells with short- and long-term LPS exposure, or inhibitors (Dexamethasome and Bay11-7082) treatment, which lead to a defective expression of TSLP and IL33.

In this study, we delineated the cellular and molecular roles of airway epithelial cells in hygiene hypothesis *in vitro*. Treatment of LPS could suppress the levels by subverting the polyI:C- and viral infection-mediated TBK1, IRF3, and NF-κB response. Therefore, the level of LPS exposure played a key role in inhibiting TSLP and IL33 expression in epithelial cells. Moreover, the DAMP inflammation factor HMGB1 increased the dsRNA-mediated proallergic cytokines expression. Blocking IRF3 and NF-κB by shRNA gene knockdown or inhibitors treatment suppressed TSLP and IL33 induction, so, in addition to TSLP and IL33, epithelium IRF3 and NF-κB activity could be targets for allergic inflammation therapy (52) (Figure 9).

Epithelial cells play key roles in bridging the innate and adaptive immune systems (15). The TLR signaling plays the crucial role in this system; however, TLR3 not only induce host inflammation response but also promote cell apoptosis (48). Physiologically, cell death is a host defense mechanism to restrict virus expanding; the exposed-viral antigen from death cells can trigger a series of immune response activation with cytokines releasing, which is usually associated with the viral pathogenic effects (53, 54). In our study, polyI:C-induced H292 cell death was inhibited by LPS treatment, which might refer that LPS-TLR4 interaction derives a negative regulation or a desensitization mechanism to provide protection against pathogen-mediated cell death (55).

Thymic stromal lymphopoietin is expressed mainly by epithelial cells and epidermal keratinocytes; other types of cells, such as mast cells, smooth muscle cells, fibroblasts, dendritic cells, trophoblasts, and cancer or cancer-associated cells also express TSLP. TSLP expression in the epidermis, epithelium, and submucosa in skin, airway and ocular tissues plays critical role in the pathogenesis of allergic disease (56, 57). IL33 is abundantly expressed in epithelial cells from tissue exposed to the environment, and in fibroblastic reticular cells of lymphoid organ. IL33 expression has been also observed in endothelial cells from blood vessels (58). The roles of TSLP and IL33 in allergy were evaluated in TSLP receptor (TSLPR)- and IL33 receptor-deficient (T1/ST2) mice, respectively; TSLPR- and T1/ST2-knockout mice showed strong Th1 responses with high levels of IL2 and IFN-γ and impaired the Th2 response (59–61). The TSLP- and IL33-mediated Th2 response was demonstrated in TSLP- and IL33 knockout mice, respectively (60, 62). By contrast, lung-specific expression of a *Tslp* transgene induced Th2-mediated airway inflammation and hyperactivity (60). Epithelial cell-derived TSLP mediates chemotactic activity in dendritic cells and airway smooth muscle cells (63, 64), which may be associated with the development of allergy. During allergic inflammation initiation, polyI:C- or dsRNA-induced TSLP and IL33 derived from epithelium are critical to activate dendritic cells to produce IL4, IL5, and IL13 and to promote Th2 responses. In turn, the positive feedback provided by IL4, IL5, and IL13 upregulates TSLP and IL-33 production in various cell types, which may aggravate allergic inflammation (Figure S7 in Supplementary Material) (10, 16, 56, 65–67). Thus, blocking TSLP and IL33 activity by antibody or signaling inhibitors may be considered for atopic disease therapy (68, 69). Alternatively, inhibition of TSLP and IL33 levels in epithelial cells may effectively attenuate allergic responses.

In this study, we demonstrated that TSLP and IL33 were induced by polyI:C and HPeV1 stimulation in H292 cells, which supported result from the previous study of primary bronchial epithelial cells (70). Nevertheless, our model also serves as a cell line-based platform for anti-allergy drug screening. We found that dexamethasone and Bay 11-7082 inhibited TSLP and IL33 expression by downregulating IRF3 and NF-κB activities. Coincidentally, the essential roles of the IRF3 and NF-κB pathways in TSLP and IL-33 induction were demonstrated in other research models [(16, 71–75), Schuijs et al. (76) #5788]. Moreover, we found that LPS-modulated IRF3 and NF-κB activation in the polyI:C or HPeV1/TLR3 axis could be the causal mechanism of hygiene hypothesis. Our findings are supported by a current study that farm dust and LPS attenuated house dust mite-mediated allergic response in respiratory epithelial cells (76).

The dose of LPS is one of the critical factors in the establishment of airway allergy. However, the used- dose of LPS *in vitro* experiments was varied. In primary cells, such as macrophages, dendrite cells, or embryonic fibroblast, 100 ng/ml–1 μg/ml of LPS is sufficient to activate innate immunity (77–80). However, in the macrophage cell line (JA774A.1) or lung carcinoma epithelial cells (A549 and BEAS-2B cells), 10–100 μg/ml of LPS was used to change cellular behaviors (40, 55). In our model, TSLP and IL33 production were downregulated in H292 cells with 30 μg/ml of LPS, short-term treatment or 0.3 μg/ml of LPS, long-term treatment. Moreover, 4–12 μg/ml of LPS also attenuate TSLP expression in human primary nasal epithelial cells. We thought that our data are not conflicting in certain with the previous reports; moreover, it also suggests that the stimulation dose of LPS and time course were critical factors in the inhibition of allergic cytokine induction.

We had tried to test our hypothesis in an experimental mouse model of allergic rhinitis; however, the animal model was extremely hard to conduct and there were several points, which are difficult to overcome in our system. First, mice always sneezed and rubbed their noses until the fluid flowed out after reagent solutions, such as LPS were dropped into their nasal cavity, so the scheduled incubation period of 1–3 h became unreliable and the stimulating dosage unreliable too. Second, when the reagent solutions stimulated the nasal cavity, we cannot differentiate whether the effects came from stimulation of the dendritic cells or epithelial cells. We thought that establishment of a proper animal model would be required for further translation approach.

Even though, the effect of LPS dose was investigated in mice reported by others, that low-dose LPS induced Th2 responses, but high-dose LPS with antigen treatment resulted in Th1 responses (29). Therefore, the level of LPS exposure can determine the skew of Th1/Th2 responses and provide a potential mechanistic explanation for epidemiological findings on LPS exposure and asthma prevalence involving the activation of antigen-containing dendritic cells (29). However, in this mouse model, only endpoint analysis was accessed; it would be of interest to investigate the immunoregulatory effect in mice receiving low-level LPS for an extended/long-term period. In addition, the physical effective dose of LPS may be varied among LPS from various bacteria (40); so, the different allergic regulation activity may be observed in mice challenged with various types and dose of bacterial LPS.

We show that short- and long-term LPS exposure had similar inhibitory effects in polyI:C/TLR3-axis-mediated TSLP and IL33 expression, with certain signaling-protein gene transcripts downregulated in H292 cells after treatment with high-dose LPS for 8 and 16 days. We also found that low-dose LPS-suppressed expression of TLR signaling genes was substantial in H292 cells treated with LPS for 2 months. Therefore, LPS silenced the TLR-mediated innate immunity, which may occur at the gene transcription level and be controlled by chromatin modification activity (81, 82). Thus, determining whether the epigenetic regulation of the host response to LPS is involved in the hygiene hypothesis would be of interest.

A recent study proposed a hypothesis about early life airway exposure to microbial pathogens and the development of asthma, which suggests that specific microbial colonization and co-infection of bacteria and viruses such as *Streptococcus* and *Corynebacterium*, respiratory syncytial virus and human rhinoviruses in infants was a strong predictor of persistent asthma developing by 5 years of age. Additionally, antibiotic treatment reduced this risk by disrupting microbial colonization (83, 84). The hypothesis that infections trigger asthma suggests an additional opinion on the hygiene hypothesis and refers to the sophisticated regulation of specific microbes and a host defense system that affect the occurrence of atopy. Therefore, our data raise important issues as to whether different LPS resources from various microbes have different inhibitory outcomes.

The LPS from *Escherichia coli* 0111:B4 was mainly used in this study, but LPS derived from other bacteria, such as *Klebsiella pneumoniae* or *Salmonella enteric*, were also tested. Although, the inhibitory effect of all these LPS on polyI:C were observed, but the implication of the pathogenic bacteria infection in allergy remains to be explored. Moreover, determining whether the inhibitory effect of LPS could be demonstrated with other bacterial components, such as peptidoglycan, teichoic acid, or phosphorylcholine in airway epithelial cells would be of interest (85).

High-mobility group protein B1 act as endogenous danger signals to promote and exacerbate the inflammatory response (37, 38, 86, 87). Given the relevance of HMGB1 as a ligand for TLRs (88, 89), we tested the activity of HMGB1 in our *in vitro* model. We found that HMGB1 itself was not able to induce TSLP and IL33 expression. However, in the polyI:C-stimulated cells, the level of TSLP and IL33 expression and IRF3 phosphorylation was enhanced by HMGB1; which is contrary to LPS treatment on the regulation of allergic inflammation. We thought that certain additional activation signaling derived from HMGB1 receptor other than TLR4, such as RAGE, might contribute to dsRNA-mediated allergic inflammation (90). Thus, it would be important to future evaluate whether HMGB1 would be a therapeutic target for allergy (91).

To the best of our knowledge, our study is the first to delineate a cellular and molecular mechanism of the hygiene hypothesis via TLR signaling in epithelial cells. In addition, we also revealed HPeV1 elicits allergic inflammation. Varying the concentrations or treatment time course of TLR4 agonist could regulate TLR3-associated allergic inflammation for a new strategy to combat allergic diseases.

## Author Contributions

T-HL, H-YK, and T-HC conceived and designed the experiments. T-HL and C-CC performed the experiments. T-HL, C-CC, H-YK, and T-HC analyzed the data. H-HS, N-CH, J-JC, and H-YK contributed reagents, materials, and analysis tools. T-HL and T-HC wrote the manuscript. All authors reviewed the manuscript.

## Conflict of Interest Statement

The authors declare that the research was conducted in the absence of any commercial or financial relationships that could be construed as a potential conflict of interest.

## References

[1] Strachan DP Hay fever, hygiene, and household size. BMJ (1989) 299(6710):1259–60. 10.1136/bmj.299.6710.1259 2513902 PMC1838109

[2] Braun-Fahrlander C. Environmental exposure to endotoxin and other microbial products and the decreased risk of childhood atopy: evaluating developments since April 2002. Curr Opin Allergy Clin Immunol (2003) 3(5):325–9. 10.1097/01.all.0000092600.10871.e0 14501429

[3] Douwes J Brooks C Pearce N Protective effects of farming on allergies and asthma: have we learnt anything since 1873? Expert Rev Clin Immunol (2009) 5(3):213–9. 10.1586/eci.09.19 20476997

[4] Riedler J Braun-Fahrlander C Eder W Schreuer M Waser M Maisch S Exposure to farming in early life and development of asthma and allergy: a cross-sectional survey. Lancet (2001) 358(9288):1129–33. 10.1016/S0140-6736(01)06252-3 11597666

[5] van Strien RT Engel R Holst O Bufe A Eder W Waser M Microbial exposure of rural school children, as assessed by levels of N-acetyl-muramic acid in mattress dust, and its association with respiratory health. J Allergy Clin Immunol (2004) 113(5):860–7. 10.1016/j.jaci.2004.01.783 15131567

[6] Kawai T Akira S. Toll-like receptors and their crosstalk with other innate receptors in infection and immunity. Immunity (2011) 34(5):637–50. 10.1016/j.immuni.2011.05.006 21616434

[7] Hammad H Chieppa M Perros F Willart MA Germain RN Lambrecht BN. House dust mite allergen induces asthma via toll-like receptor 4 triggering of airway structural cells. Nat Med (2009) 15(4):410–6. 10.1038/nm.1946 19330007 PMC2789255

[8] Zarember KA Godowski PJ. Tissue expression of human toll-like receptors and differential regulation of toll-like receptor mRNAs in leukocytes in response to microbes, their products, and cytokines. J Immunol (2002) 168(2):554–61. 10.4049/jimmunol.168.2.554 11777946

[9] Lay MK Cespedes PF Palavecino CE Leon MA Diaz RA Salazar FJ Human metapneumovirus infection activates the TSLP pathway that drives excessive pulmonary inflammation and viral replication in mice. Eur J Immunol (2015) 45(6):1680–95. 10.1002/eji.201445021 25763996

[10] Soumelis V Reche PA Kanzler H Yuan W Edward G Homey B Human epithelial cells trigger dendritic cell mediated allergic inflammation by producing TSLP. Nat Immunol (2002) 3(7):673–80. 10.1038/ni805 12055625

[11] Schmitz J Owyang A Oldham E Song Y Murphy E McClanahan TK IL-33, an interleukin-1-like cytokine that signals via the IL-1 receptor-related protein ST2 and induces T helper type 2-associated cytokines. Immunity (2005) 23(5):479–90. 10.1016/j.immuni.2005.09.015 16286016

[12] Bogiatzi SI Fernandez I Bichet JC Marloie-Provost MA Volpe E Sastre X Cutting edge: proinflammatory and Th2 cytokines synergize to induce thymic stromal lymphopoietin production by human skin keratinocytes. J Immunol (2007) 178(6):3373–7. 10.4049/jimmunol.178.6.3373 17339431

[13] Islam SA Luster AD. T cell homing to epithelial barriers in allergic disease. Nat Med (2012) 18(5):705–15. 10.1038/nm.2760 22561834 PMC3863331

[14] Salazar F Ghaemmaghami AM. Allergen recognition by innate immune cells: critical role of dendritic and epithelial cells. Front Immunol (2013) 4:356. 10.3389/fimmu.2013.00356 24204367 PMC3816228

[15] Kato A Schleimer RP. Beyond inflammation: airway epithelial cells are at the interface of innate and adaptive immunity. Curr Opin Immunol (2007) 19(6):711–20. 10.1016/j.coi.2007.08.004 17928212 PMC2196222

[16] Kato A Favoreto S Jr Avila PC Schleimer RP. TLR3- and Th2 cytokine-dependent production of thymic stromal lymphopoietin in human airway epithelial cells. J Immunol (2007) 179(2):1080–7. 10.4049/jimmunol.179.2.1080 17617600 PMC2220044

[17] Lee HC Headley MB Loo YM Berlin A Gale M Jr Debley JS Thymic stromal lymphopoietin is induced by respiratory syncytial virus-infected airway epithelial cells and promotes a type 2 response to infection. J Allergy Clin Immunol (2012) 130(5):1187.e–96.e. 10.1016/j.jaci.2012.07.031 22981788 PMC4284103

[18] Perez GF Pancham K Huseni S Preciado D Freishtat RJ Colberg-Poley AM Rhinovirus infection in young children is associated with elevated airway TSLP levels. Eur Respir J (2014) 44(4):1075–8. 10.1183/09031936.00049214 24969655 PMC4183718

[19] Jackson DJ Makrinioti H Rana BM Shamji BW Trujillo-Torralbo MB Footitt J IL-33-dependent type 2 inflammation during rhinovirus-induced asthma exacerbations in vivo. Am J Respir Crit Care Med (2014) 190(12):1373–82. 10.1164/rccm.201406-1039OC 25350863 PMC4299647

[20] Kamekura R Kojima T Takano K Go M Sawada N Himi T. The role of IL-33 and its receptor ST2 in human nasal epithelium with allergic rhinitis. Clin Exp Allergy (2012) 42(2):218–28. 10.1111/j.1365-2222.2011.03867.x 22233535

[21] Nateri AS Hughes PJ Stanway G. In vivo and in vitro identification of structural and sequence elements of the human parechovirus 5′ untranslated region required for internal initiation. J Virol (2000) 74(14):6269–77. 10.1128/JVI.74.14.6269-6277.2000 10864636 PMC112132

[22] Birenbaum E Handsher R Kuint J Dagan R Raichman B Mendelson E Echovirus type 22 outbreak associated with gastro-intestinal disease in a neonatal intensive care unit. Am J Perinatol (1997) 14(8):469–73. 10.1055/s-2007-994182 9376008

[23] Berkovich S Pangan J Recoveries of virus from premature infants during outbreaks of respiratory disease: the relation of ECHO virus type 22 to disease of the upper and lower respiratory tract in the premature infant. Bull N Y Acad Med (1968) 44(4):377–87. 5241248 PMC1750138

[24] Benschop KS Schinkel J Minnaar RP Pajkrt D Spanjerberg L Kraakman HC Human parechovirus infections in Dutch children and the association between serotype and disease severity. Clin Infect Dis (2006) 42(2):204–10. 10.1086/498905 16355330

[25] Groskreutz DJ Monick MM Powers LS Yarovinsky TO Look DC Hunninghake GW. Respiratory syncytial virus induces TLR3 protein and protein kinase R, leading to increased double-stranded RNA responsiveness in airway epithelial cells. J Immunol (2006) 176(3):1733–40. 10.4049/jimmunol.176.3.1733 16424203

[26] Kinoshita H Takai T Le TA Kamijo S Wang XL Ushio H Cytokine milieu modulates release of thymic stromal lymphopoietin from human keratinocytes stimulated with double-stranded RNA. J Allergy Clin Immunol (2009) 123(1):179–86. 10.1016/j.jaci.2008.10.008 19056108

[27] Arshad MI Patrat-Delon S Piquet-Pellorce C L’Helgoualc’h A Rauch M Genet V Pathogenic mouse hepatitis virus or poly(I:C) induce IL-33 in hepatocytes in murine models of hepatitis. PLoS One (2013) 8(9):e74278. 10.1371/journal.pone.0074278 24058536 PMC3772926

[28] Oriss TB Ostroukhova M Seguin-Devaux C Dixon-McCarthy B Stolz DB Watkins SC Dynamics of dendritic cell phenotype and interactions with CD4+ T cells in airway inflammation and tolerance. J Immunol (2005) 174(2):854–63. 10.4049/jimmunol.174.2.854 15634907

[29] Eisenbarth SC Piggott DA Huleatt JW Visintin I Herrick CA Bottomly K. Lipopolysaccharide-enhanced, toll-like receptor 4-dependent T helper cell type 2 responses to inhaled antigen. J Exp Med (2002) 196(12):1645–51. 10.1084/jem.20021340 12486107 PMC2196061

[30] Delayre-Orthez C Becker J de Blay F Frossard N Pons F. Exposure to endotoxins during sensitization prevents further endotoxin-induced exacerbation of airway inflammation in a mouse model of allergic asthma. Int Arch Allergy Immunol (2005) 138(4):298–304. 10.1159/000088867 16220006

[31] Ray A Chakraborty K Ray P. Immunosuppressive MDSCs induced by TLR signaling during infection and role in resolution of inflammation. Front Cell Infect Microbiol (2013) 3:52. 10.3389/fcimb.2013.00052 24066282 PMC3776133

[32] Haapakoski R Karisola P Fyhrquist N Savinko T Lehtimaki S Wolff H Toll-like receptor activation during cutaneous allergen sensitization blocks development of asthma through IFN-gamma-dependent mechanisms. J Invest Dermatol (2013) 133(4):964–72. 10.1038/jid.2012.356 23151845

[33] Chang JT Yang CS Chen YS Chen BC Chiang AJ Chang YH Genome and infection characteristics of human parechovirus type 1: the interplay between viral infection and type I interferon antiviral system. PLoS One (2015) 10(2):e0116158. 10.1371/journal.pone.0116158 25646764 PMC4380134

[34] Kang R Chen R Zhang Q Hou W Wu S Cao L HMGB1 in health and disease. Mol Aspects Med (2014) 40:1–116. 10.1016/j.mam.2014.05.001 25010388 PMC4254084

[35] Shim EJ Chun E Lee HS Bang BR Kim TW Cho SH The role of high-mobility group box-1 (HMGB1) in the pathogenesis of asthma. Clin Exp Allergy (2012) 42(6):958–65. 10.1111/j.1365-2222.2012.03998.x 22909167

[36] Hosakote YM Brasier AR Casola A Garofalo RP Kurosky A. RSV infection triggers epithelial HMGB1 release as a damage-associated molecular pattern promoting a monocytic inflammatory response. J Virol (2016). 10.1128/JVI.01279-16 27535058 PMC5068515

[37] Yu M Wang H Ding A Golenbock DT Latz E Czura CJ HMGB1 signals through toll-like receptor (TLR) 4 and TLR2. Shock (2006) 26(2):174–9. 10.1097/01.shk.0000225404.51320.82 16878026

[38] Piccinini AM Midwood KS. DAMPening inflammation by modulating TLR signalling. Mediators Inflamm (2010) 2010:672395. 10.1155/2010/672395 20706656 PMC2913853

[39] Werner U Kissel T. In-vitro cell culture models of the nasal epithelium: a comparative histochemical investigation of their suitability for drug transport studies. Pharm Res (1996) 13(7):978–88. 10.1023/A:1016020505313 8842033

[40] Koyama S Sato E Nomura H Kubo K Miura M Yamashita T The potential of various lipopolysaccharides to release monocyte chemotactic activity from lung epithelial cells and fibroblasts. Eur Respir J (1999) 14(3):545–52. 10.1034/j.1399-3003.1999.14c11.x 10543273

[41] Chang TH Liao CL Lin YL. Flavivirus induces interferon-beta gene expression through a pathway involving RIG-I-dependent IRF-3 and PI3K-dependent NF-kappaB activation. Microbes Infect (2006) 8(1):157–71. 10.1016/j.micinf.2005.06.014 16182584

[42] Cook JA Mitchell JB Viability measurements in mammalian cell systems. Anal Biochem (1989) 179(1):1–7. 10.1016/0003-2697(89)90191-7 2667390

[43] Chou CP Huang NC Jhuang SJ Pan HB Peng NJ Cheng JT Ubiquitin-conjugating enzyme UBE2C is highly expressed in breast microcalcification lesions. PLoS One (2014) 9(4):e93934. 10.1371/journal.pone.0093934 24699941 PMC3974821

[44] Ponchel F Toomes C Bransfield K Leong FT Douglas SH Field SL Real-time PCR based on SYBR-Green I fluorescence: an alternative to the TaqMan assay for a relative quantification of gene rearrangements, gene amplifications and micro gene deletions. BMC Biotechnol (2003) 3:18. 10.1186/1472-6750-3-18 14552656 PMC270040

[45] Chang TH Yoshimi R Ozato K Tripartite motif (TRIM) 12c, a mouse homolog of TRIM5, is a ubiquitin ligase that stimulates type I IFN and NF-kappaB pathways along with TNFR-associated factor 6. J Immunol (2015) 195(11):5367–79. 10.4049/jimmunol.1402064 26503954 PMC4654225

[46] Wang LF Lin YS Huang NC Yu CY Tsai WL Chen JJ Hydroxychloroquine-inhibited dengue virus is associated with host defense machinery. J Interferon Cytokine Res (2015) 35(3):143–56. 10.1089/jir.2014.0038 25321315 PMC4350140

[47] Vroling AB Jonker MJ Breit TM Fokkens WJ van Drunen CM. Comparison of expression profiles induced by dust mite in airway epithelia reveals a common pathway. Allergy (2008) 63(4):461–7. 10.1111/j.1398-9995.2007.01621.x 18315734

[48] Sun R Zhang Y Lv Q Liu B Jin M Zhang W Toll-like receptor 3 (TLR3) induces apoptosis via death receptors and mitochondria by up-regulating the transactivating p63 isoform alpha (TAP63alpha). J Biol Chem (2011) 286(18):15918–28. 10.1074/jbc.M110.178798 21367858 PMC3091201

[49] Vroling AB Fokkens WJ van Drunen CM. How epithelial cells detect danger: aiding the immune response. Allergy (2008) 63(9):1110–23. 10.1111/j.1398-9995.2008.01785.x 18699929

[50] Scheinman RI Gualberto A Jewell CM Cidlowski JA Baldwin AS Jr. Characterization of mechanisms involved in transrepression of NF-kappa B by activated glucocorticoid receptors. Mol Cell Biol (1995) 15(2):943–53. 10.1128/MCB.15.2.943 7823959 PMC231982

[51] Strickson S Campbell DG Emmerich CH Knebel A Plater L Ritorto MS The anti-inflammatory drug BAY 11-7082 suppresses the MyD88-dependent signalling network by targeting the ubiquitin system. Biochem J (2013) 451(3):427–37. 10.1042/BJ20121651 23441730 PMC3685219

[52] Szelag M Piaszyk-Borychowska A Plens-Galaska M Wesoly J Bluyssen HA. Targeted inhibition of STATs and IRFs as a potential treatment strategy in cardiovascular disease. Oncotarget (2016). 10.18632/oncotarget.9195 27166190 PMC5217051

[53] Miedema F Hazenberg MD Tesselaar K van Baarle D de Boer RJ Borghans JA. Immune activation and collateral damage in AIDS pathogenesis. Front Immunol (2013) 4:298. 10.3389/fimmu.2013.00298 24133492 PMC3783946

[54] Falasca L Agrati C Petrosillo N Di Caro A Capobianchi MR Ippolito G Molecular mechanisms of Ebola virus pathogenesis: focus on cell death. Cell Death Differ (2015) 22(8):1250–9. 10.1038/cdd.2015.67 26024394 PMC4495366

[55] Ruckdeschel K Richter K. Lipopolysaccharide desensitization of macrophages provides protection against *Yersinia enterocolitica*-induced apoptosis. Infect Immun (2002) 70(9):5259–64. 10.1128/IAI.70.9.5259-5264.2002 12183578 PMC128233

[56] Takai T. TSLP expression: cellular sources, triggers, and regulatory mechanisms. Allergol Int (2012) 61(1):3–17. 10.2332/allergolint.11-RAI-0395 22270071

[57] Liu YJ. Thymic stromal lymphopoietin: master switch for allergic inflammation. J Exp Med (2006) 203(2):269–73. 10.1084/jem.20051745 16432252 PMC2118215

[58] Cayrol C Girard JP. IL-33: an alarmin cytokine with crucial roles in innate immunity, inflammation and allergy. Curr Opin Immunol (2014) 31:31–7. 10.1016/j.coi.2014.09.004 25278425

[59] Al-Shami A Spolski R Kelly J Keane-Myers A Leonard WJ. A role for TSLP in the development of inflammation in an asthma model. J Exp Med (2005) 202(6):829–39. 10.1084/jem.20050199 16172260 PMC2212950

[60] Zhou B Comeau MR De Smedt T Liggitt HD Dahl ME Lewis DB Thymic stromal lymphopoietin as a key initiator of allergic airway inflammation in mice. Nat Immunol (2005) 6(10):1047–53. 10.1038/ni1247 16142237

[61] Townsend MJ Fallon PG Matthews DJ Jolin HE McKenzie AN. T1/ST2-deficient mice demonstrate the importance of T1/ST2 in developing primary T helper cell type 2 responses. J Exp Med (2000) 191(6):1069–76. 10.1084/jem.191.6.1069 10727469 PMC2193113

[62] Jang S Morris S Lukacs NW TSLP promotes induction of Th2 differentiation but is not necessary during established allergen-induced pulmonary disease. PLoS One (2013) 8(2):e56433 10.1371/journal.pone.0056433 23437132 PMC3577905

[63] Fernandez MI Heuze ML Martinez-Cingolani C Volpe E Donnadieu MH Piel M The human cytokine TSLP triggers a cell-autonomous dendritic cell migration in confined environments. Blood (2011) 118(14):3862–9. 10.1182/blood-2010-12-323089 21772055

[64] Redhu NS Shan L Movassagh H Gounni AS. Thymic stromal lymphopoietin induces migration in human airway smooth muscle cells. Sci Rep (2013) 3:2301. 10.1038/srep02301 23892442 PMC3725475

[65] Ryu WI Lee H Kim JH Bae HC Ryu HJ Son SW. IL-33 induces Egr-1-dependent TSLP expression via the MAPK pathways in human keratinocytes. Exp Dermatol (2015) 24(11):857–63. 10.1111/exd.12788 26120956

[66] Besnard AG Togbe D Guillou N Erard F Quesniaux V Ryffel B. IL-33-activated dendritic cells are critical for allergic airway inflammation. Eur J Immunol (2011) 41(6):1675–86. 10.1002/eji.201041033 21469105

[67] Rank MA Kobayashi T Kozaki H Bartemes KR Squillace DL Kita H. IL-33-activated dendritic cells induce an atypical TH2-type response. J Allergy Clin Immunol (2009) 123(5):1047–54. 10.1016/j.jaci.2009.02.026 19361843 PMC2711963

[68] Gauvreau GM O’Byrne PM Boulet LP Wang Y Cockcroft D Bigler J Effects of an anti-TSLP antibody on allergen-induced asthmatic responses. N Engl J Med (2014) 370(22):2102–10. 10.1056/NEJMoa1402895 24846652

[69] Nabe T. Interleukin (IL)-33: new therapeutic target for atopic diseases. J Pharmacol Sci (2014) 126(2):85–91. 10.1254/jphs.14R12CP 25213717

[70] Brandelius A Yudina Y Calven J Bjermer L Andersson M Persson C dsRNA-induced expression of thymic stromal lymphopoietin (TSLP) in asthmatic epithelial cells is inhibited by a small airway relaxant. Pulm Pharmacol Ther (2011) 24(1):59–66. 10.1016/j.pupt.2010.10.004 20951221

[71] Cultrone A de Wouters T Lakhdari O Kelly D Mulder I Logan E The NF-kappaB binding site located in the proximal region of the TSLP promoter is critical for TSLP modulation in human intestinal epithelial cells. Eur J Immunol (2013) 43(4):1053–62. 10.1002/eji.201142340 23310954

[72] Vu AT Chen X Xie Y Kamijo S Ushio H Kawasaki J Extracellular double-stranded RNA induces TSLP via an endosomal acidification- and NF-kappaB-dependent pathway in human keratinocytes. J Invest Dermatol (2011) 131(11):2205–12. 10.1038/jid.2011.185 21716324

[73] Lee HC Ziegler SF. Inducible expression of the proallergic cytokine thymic stromal lymphopoietin in airway epithelial cells is controlled by NFkappaB. Proc Natl Acad Sci U S A (2007) 104(3):914–9. 10.1073/pnas.0607305104 17213320 PMC1783414

[74] Negishi H Miki S Sarashina H Taguchi-Atarashi N Nakajima A Matsuki K Essential contribution of IRF3 to intestinal homeostasis and microbiota-mediated Tslp gene induction. Proc Natl Acad Sci U S A (2012) 109(51):21016–21. 10.1073/pnas.1219482110 23213237 PMC3529020

[75] Polumuri SK Jayakar GG Shirey KA Roberts ZJ Perkins DJ Pitha PM Transcriptional regulation of murine IL-33 by TLR and non-TLR agonists. J Immunol (2012) 189(1):50–60. 10.4049/jimmunol.1003554 22634618 PMC3437667

[76] Schuijs MJ Willart MA Vergote K Gras D Deswarte K Ege MJ Farm dust and endotoxin protect against allergy through A20 induction in lung epithelial cells. Science (2015) 349(6252):1106–10. 10.1126/science.aac6623 26339029

[77] Tsujimura H Tamura T Gongora C Aliberti J Reis e Sousa C Sher A ICSBP/IRF-8 retrovirus transduction rescues dendritic cell development in vitro. Blood (2003) 101(3):961–9. 10.1182/blood-2002-05-1327 12393459

[78] Lombardo E Alvarez-Barrientos A Maroto B Bosca L Knaus UG. TLR4-mediated survival of macrophages is MyD88 dependent and requires TNF-alpha autocrine signalling. J Immunol (2007) 178(6):3731–9. 10.4049/jimmunol.178.6.3731 17339471

[79] Tailor P Tamura T Morse HC III Ozato K. The BXH2 mutation in IRF8 differentially impairs dendritic cell subset development in the mouse. Blood (2008) 111(4):1942–5. 10.1182/blood-2007-07-100750 18055870 PMC2234043

[80] Yoshimi R Chang TH Wang H Atsumi T Morse HC III Ozato K. Gene disruption study reveals a nonredundant role for TRIM21/Ro52 in NF-kappaB-dependent cytokine expression in fibroblasts. J Immunol (2009) 182(12):7527–38. 10.4049/jimmunol.0804121 19494276 PMC2803686

[81] Arbibe L Kim DW Batsche E Pedron T Mateescu B Muchardt C An injected bacterial effector targets chromatin access for transcription factor NF-kappaB to alter transcription of host genes involved in immune responses. Nat Immunol (2007) 8(1):47–56. 10.1038/ni1423 17159983

[82] Foster SL Hargreaves DC Medzhitov R. Gene-specific control of inflammation by TLR-induced chromatin modifications. Nature (2007) 447(7147):972–8. 10.1038/nature05836 17538624

[83] Teo SM Mok D Pham K Kusel M Serralha M Troy N The infant nasopharyngeal microbiome impacts severity of lower respiratory infection and risk of asthma development. Cell Host Microbe (2015) 17(5):704–15. 10.1016/j.chom.2015.03.008 25865368 PMC4433433

[84] Webley WC Aldridge KL. Infectious asthma triggers: time to revise the hygiene hypothesis? Trends Microbiol (2015) 23(7):389–91. 10.1016/j.tim.2015.05.006 26070971

[85] Patel PS Kearney JF. Neonatal exposure to pneumococcal phosphorylcholine modulates the development of house dust mite allergy during adult life. J Immunol (2015) 194(12):5838–50. 10.4049/jimmunol.1500251 25957171 PMC4456637

[86] Lebeer S Vanderleyden J De Keersmaecker SC. Host interactions of probiotic bacterial surface molecules: comparison with commensals and pathogens. Nat Rev Microbiol (2010) 8(3):171–84. 10.1038/nrmicro2297 20157338

[87] Hreggvidsdottir HS Ostberg T Wahamaa H Schierbeck H Aveberger AC Klevenvall L The alarmin HMGB1 acts in synergy with endogenous and exogenous danger signals to promote inflammation. J Leukoc Biol (2009) 86(3):655–62. 10.1189/jlb.0908548 19564572

[88] Bauer EM Shapiro R Billiar TR Bauer PM. High mobility group box 1 inhibits human pulmonary artery endothelial cell migration via a toll-like receptor 4- and interferon response factor 3-dependent mechanism(s). J Biol Chem (2013) 288(2):1365–73. 10.1074/jbc.M112.434142 23148224 PMC3543019

[89] Yanai H Ban T Wang Z Choi MK Kawamura T Negishi H HMGB proteins function as universal sentinels for nucleic-acid-mediated innate immune responses. Nature (2009) 462(7269):99–103. 10.1038/nature08512 19890330

[90] Liang Y Hou C Kong J Wen H Zheng X Wu L HMGB1 binding to receptor for advanced glycation end products enhances inflammatory responses of human bronchial epithelial cells by activating p38 MAPK and ERK1/2. Mol Cell Biochem (2015) 405(1–2):63–71. 10.1007/s11010-015-2396-0 25862459

[91] Mollica L De Marchis F Spitaleri A Dallacosta C Pennacchini D Zamai M Glycyrrhizin binds to high-mobility group box 1 protein and inhibits its cytokine activities. Chem Biol (2007) 14(4):431–41. 10.1016/j.chembiol.2007.03.007 17462578

