# Supplementary material for: Lipopolysaccharide Attenuates Induction of Proallergic Cytokines, Thymic Stromal Lymphopoietin, and Interleukin 33 in Respiratory Epithelial Cells Stimulated with PolyI:C and Human Parechovirus
Source: Front Immunol. 2016 Oct 25;7:440. doi: 10.3389/fimmu.2016.00440 (PMC5078322; doi:10.3389/fimmu.2016.00440)
Supplement: Supplementary file 1 [file Data_Sheet_1.PDF]

Supplementary Material

Lipopolysaccharide attenuates induction of pro-allergic cytokines, thymic stromal lymphopoietin and interleukin 33, in respiratory epithelial cells stimulated with polyI:C and human parechovirus

Tsang-Hsiung Lin, Chih-Chi Cheng, Hsing-Hao Su, Nan-Chieh Huang, Jih-Jung Chen, Hong-Yo Kang, Tsung-Hsien Chang

Table S1. qPCR primer sequences used in this study

Genes	Primer sequences
TSLP	F- GCAGGCACCCTCTCACTCA<br>R-TGCCCTTCCCACTGGTGTT
IL33	F- GGCTGCATCACGTTGTACTTTG<br>R- CCACATGTAGATTAGGCCTCAGATT
IL25	F- CGCCACCCAGAGTCCTGTAG<br>R- CCGGTTCAAGTCTCTGTCCAA
NF-κB p65	F- CCATGGCTGAAGGAA ACCA<br>R- CAGACCAAACCCCTTCTGGAT
NEMO	F- GCGCTCTATCGAGGTCGTTAA<br>R- CAAGGGCTAGCTGCCAAACT
IRAK1	F- GCCCCTTTCCGTTTTGCT<br>R- GATCTTGAGCTCCTCCGAGAAG
NOD1	F- TGGTCACCGCTCTTTTCATTG<br>R- TATTCAAGAAGCTTGGCACCAA
NOD2	F- GGCTCTGTATTTGCGCGATAA<br>R- GCAATTGCTCGCAGTGAAGA
RIP1	F- AATGGCGGCACCCTCTACTA<br>R- CTTTGCGTTGACGTCATTCAG
MAP3K7	F- GCCGCAACCACAGCCTAT<br>R- TGCCAAATGAAGCAGTTTTACG
TRAF6	F- TCAGTACTTTTGGTTGCCATGAA<br>R- AGCCTGGGCCAACATTCTC
TLR3	F- CCTGGTTTGTTAATTGGATTAACGA<br>R- TGAGGTGGAGTGTTGCAAAGG
GAPDH	F- TGCACCACCAACTGCTTAGC<br>R- GGCATGGACTGTGGTCAT

HPeV1 VP1 (+)	F-CGACACATCACAAAACAGGGTTA<br>R-ATCTGTTCTCCTGCTGGAATTGTAA
HPeV1 VP1 (-)	F-ACCCTGTTTTGTGATGTGTCGTA<br>R-TGTTTTGTTCTTGGCTGGAAGA

Figure S1

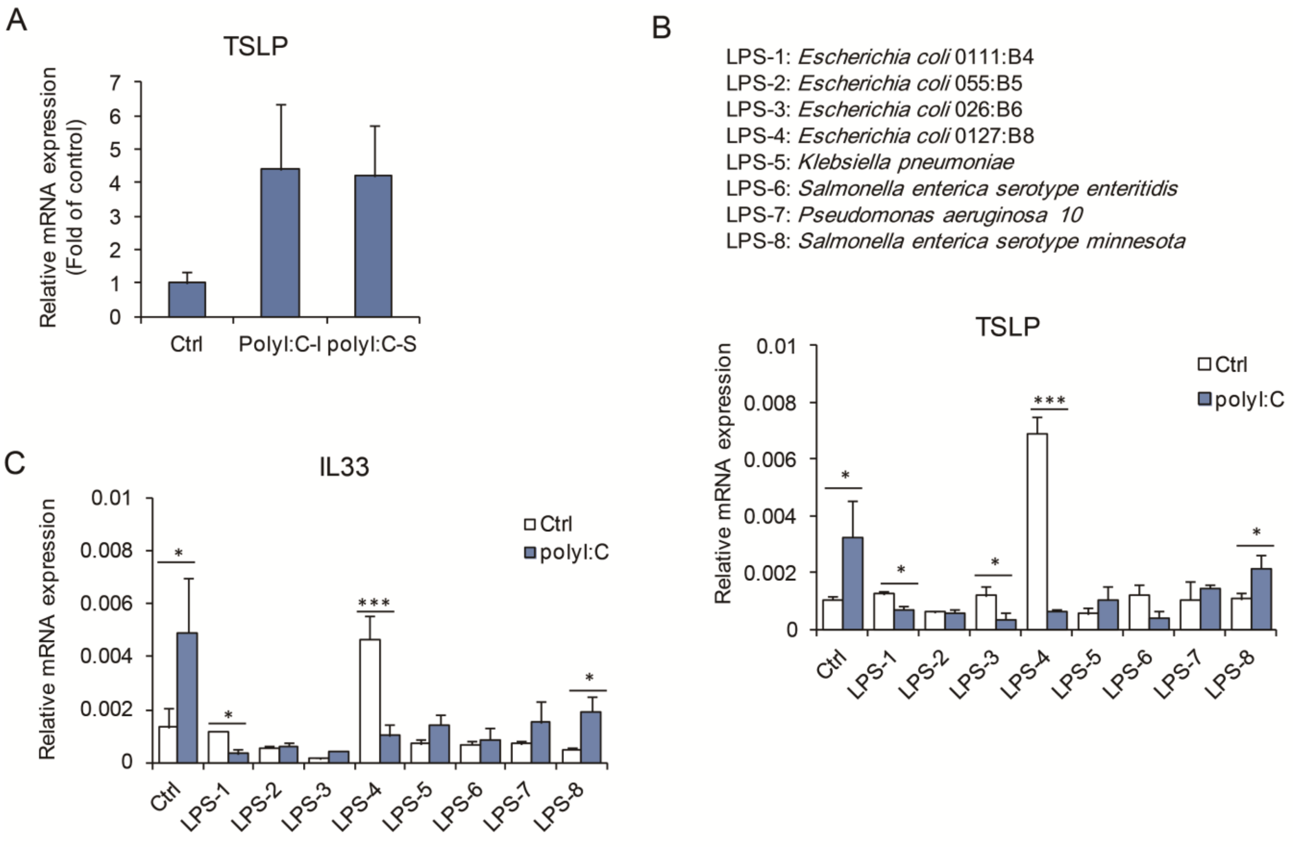

Figure S1. Comparison of 2 polyI:C and 8 LPS products on the TSLP induction. (A) RT-qPCR analysis of thymic stromal lymphopoietin (TSLP), interleukin 33 (IL33) expression in H292 cells stimulated with 2 products of polyI:C (2.5 μg) for 3 h. PolyI:C-I, polyIC from InvivoGen; polyI:C-S, polyI:C from Sigma-Aldrich. **(B and C)** H292 cells were pretreated with 8 different LPS (30 μg/ml) from indicated various bacteria strains for 2 h, then stimulated with polyI:C for 3 h. The expression of TSLP and IL33 were monitored by RT-qPCR and normalized to the internal control GAPDH; fold induction over controls is presented. Data of RT-qPCR values are mean ± SD from 3 independent experiments. Two- tailed student t-test, *P<0.05, ***P<0.001 compared to controls; #P<0.05.

Figure S2

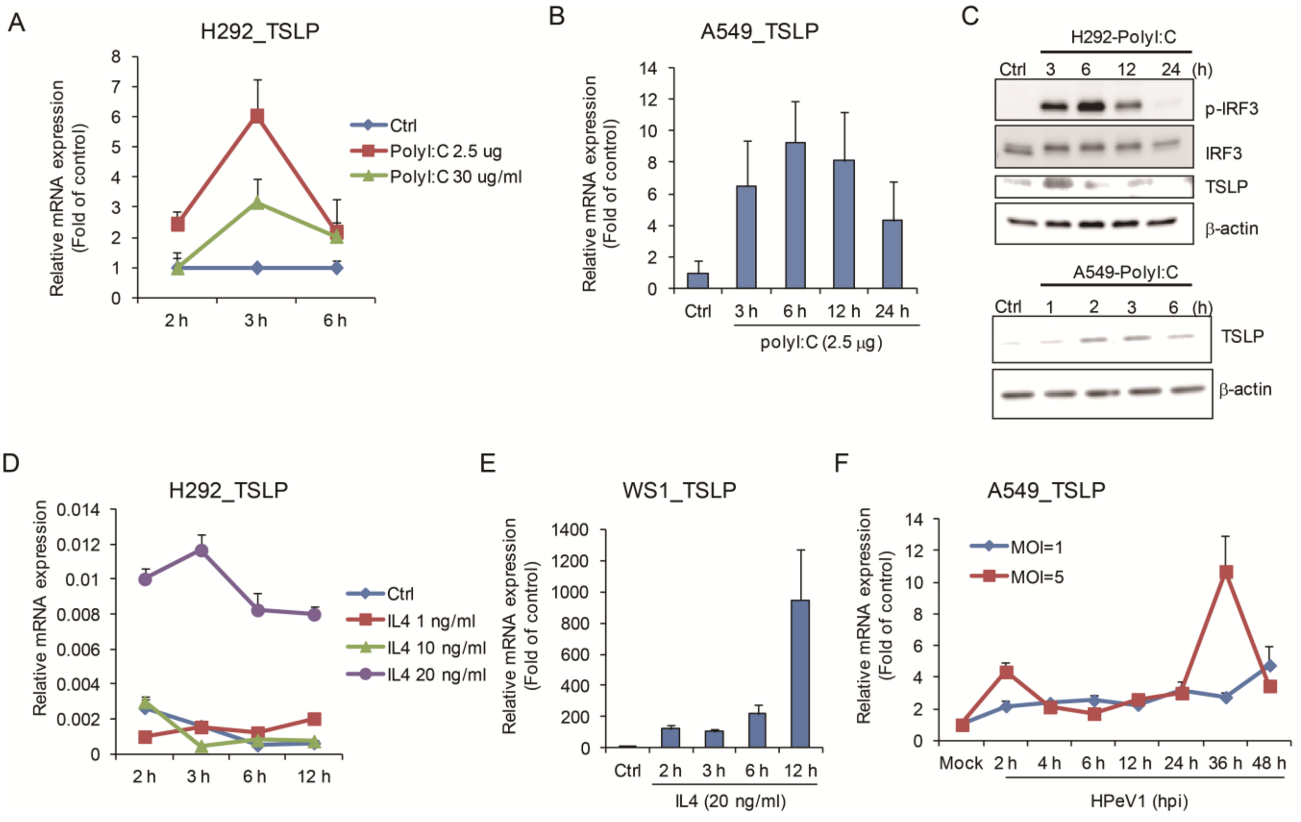

Figure S2. The TSLP induction in various cell types. (A) TSLP mRNA expression was measured in H292 cells stimulated with polyI:C by transfection (2.5 μg) or by directly adding into medium (30 μg/ml). **(B)** TSLP mRNA expression was determined in polyI:C stimulated A549 cells at different times. **(C)** Immunoblotting of IRF3 and TSLP in H292 cells (upper panels) and A549 cells (lower panels) stimulated with polyI:C (2.5 μg). **(D)** RT-PCR analysis of TSLP expression in H292 cells stimulated with IL4 (1 ng/ml – 20 ng/ml) at indicated times. **(E)** TSLP mRNA was measured in IL-4 (20 ng/ml) stimulate WS-1 cells. **(F)** A549 cells (1×10^6) in 6-well plates were infected by HPeV1at MOI= 1 or 5 for various times, and then the TSLP mRNA level was determined. Data of RT-qPCR values are mean ± SD from 3 independent experiments.

Figure S3.

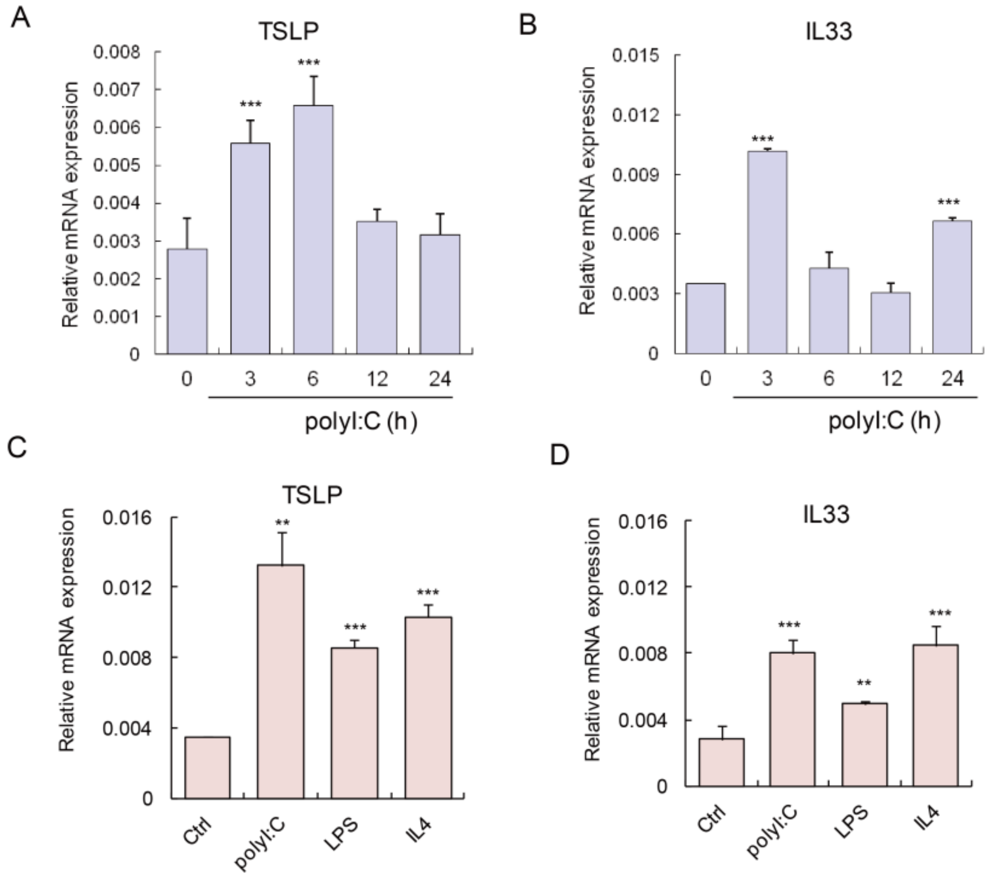

Figure S3. TSLP and IL33 induction in NL-20 cells. (A and B) RT-qPCR analysis of TSLP and IL33 expression in polyI:C (2.5 μg) stimulated NL-20 cell at indicated times. **(C and D)** NL-20 cells were treated with polyI:C (2.5 μg), LPS (30 μg/ml) or IL4 (20 ng/ml) for 3 h, and TSLP and IL33 mRNA expression was analyzed by RT-qPCR. Data are mean ± SD, ***P<0.001 compared to controls.

Figure S4

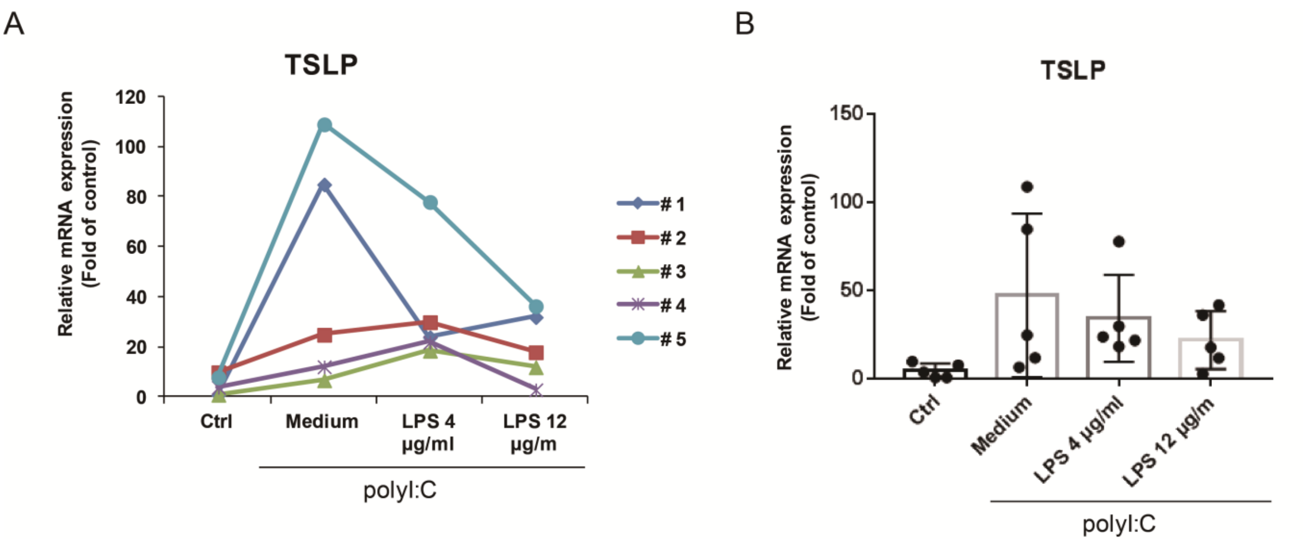

Figure S4. LPS down-regulates the TSLP and IL33 expression in human primary nasal epithelial cells. (A) Human primary nasal epithelial cells were isolated form 3 donors, and 5 independent experiments (#1~ # 5) were conducted. The cells were pretreated with LPS (4 and 12 μg/ml) for 2 h, then incubated with polyI:C (20 μg/ml) contained medium for 3 h. The expression of TSLP was monitored by RT-qPCR; fold induction over controls is presented. **(B)** The merged data from 5 experiments (A) is shown.

Figure S5

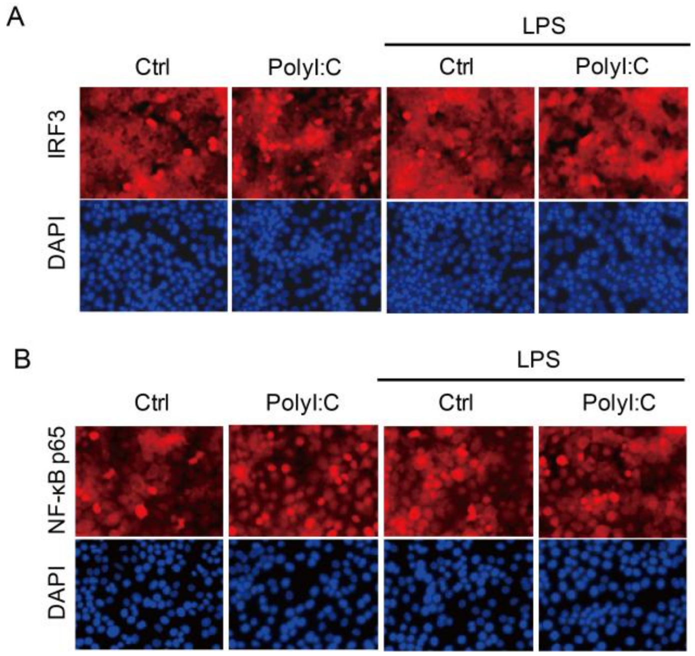

Figure S5. IRF3 and NFκB p65 nuclear translocation assay. **(A and B)** The nuclear translocation of IRF3 and NF-κB p65 was measured by immunofluorescence assay in H292 cells treated with LPS for 2 h, then polyI:C stimulation for 3 h. Red fluorescence indicates the location of IRF3 and NFκB p65, and DAPI staining (blue color) show cell nuclei.

Figure S6

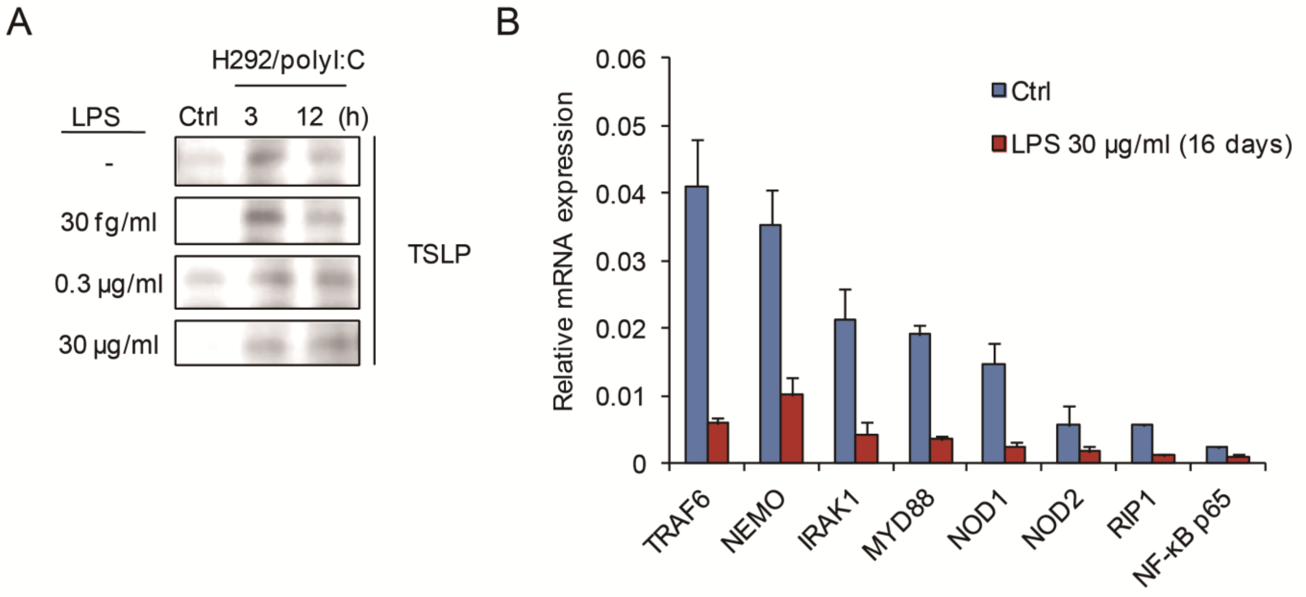

Figure S6. The TSLP protein and signaling gene expression in H292 cells with long-term LPS treatment. (A). H292 cells were treated with various doses of LPS for 8 days, then stimulated with polyI:C (2.5 µg) for 3 h. TSLP protein expression was analyzed by immunoblotting. **(B)** RT-qPCR analysis of TLR signaling gene expression in H292 cells with long-term LPS (30 µg/ml) treatment for 16 days.

Figure S7

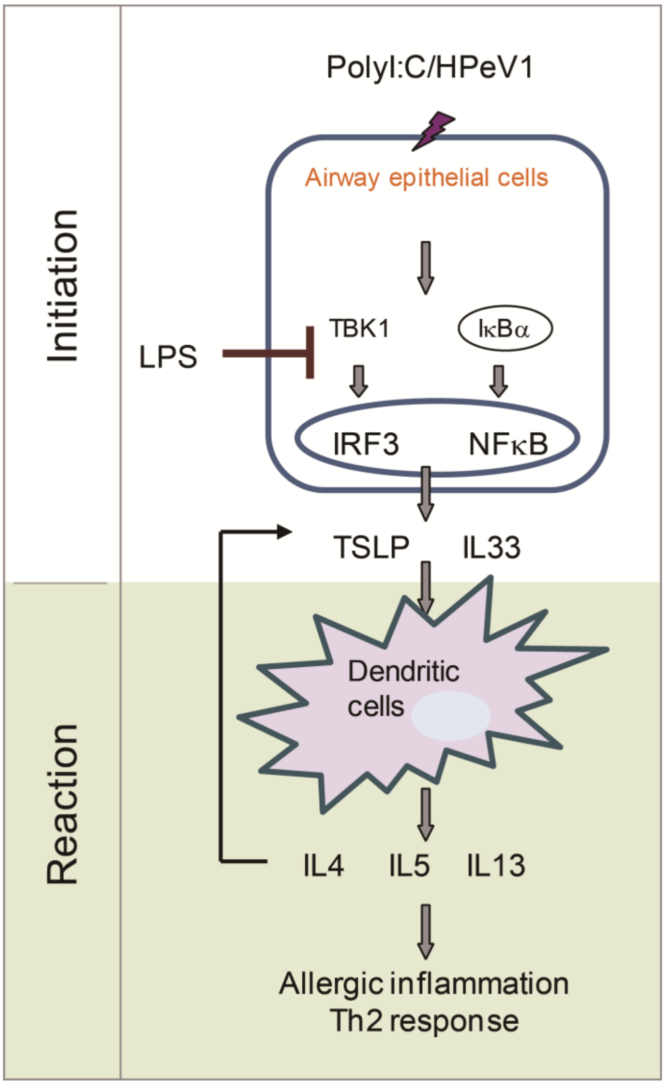

Figure S7 . Epithelial cells support the hygiene hypothesis by regulating TLR signaling pathways in response to polyI:C or HPeV1 by varying the LPS concentration. Epithelial-derived TSLP and IL33 determine the dendritic cell-mediated T helper 2 (Th2) cell response. PolyI:C and HPeV1 induce TSLP and IL33 expression via IRF3 and NFκB in air epithelial cells, which promote dendritic cells to express IL4, IL5 and IL13 and trigger allergic and Th2 responses. In addition, IL4, IL5 and IL13 can further amplify TSLP and IL33 expression in airway epithelial cells. The IRF3 and NFκB activities are downregulated by LPS in airway epithelial cells , for low expression of TSLP and IL-33.
